# Postcardiotomy extracorporeal membrane oxygenation after elective, urgent, and emergency cardiac operations: Insights from the PELS observational study

**DOI:** 10.1016/j.xjon.2025.01.018

**Published:** 2025-02-13

**Authors:** Silvia Mariani, Alvaro Perazzo, Maria Elena De Piero, Bas C.T. van Bussel, Michele Di Mauro, Dominik Wiedemann, Sven Lehmann, Matteo Pozzi, Antonio Loforte, Udo Boeken, Robertas Samalavicius, Karl Bounader, Xiaotong Hou, Jeroen J.H. Bunge, Kogulan Sriranjan, Leonardo Salazar, Bart Meyns, Michael A. Mazzeffi, Sacha Matteucci, Sandro Sponga, Graeme MacLaren, Claudio Russo, Francesco Formica, Pranya Sakiyalak, Antonio Fiore, Daniele Camboni, Giuseppe Maria Raffa, Rodrigo Diaz, I-wen Wang, Jae-Seung Jung, Jan Belohlavek, Vin Pellegrino, Giacomo Bianchi, Matteo Pettinari, Alessandro Barbone, José P. Garcia, Kiran Shekar, Glenn Whitman, Roberto Lorusso, PELS Investigators

**Affiliations:** a Maastricht University Medical Center, Cardiovascular Research Institute Maastricht, Maastricht, The Netherlands; b Cardiac Surgery Unit, Cardio-thoracic and Vascular Department, Fondazione IRCCS San Gerardo dei Tintori, Monza, Italy; c Transplant Center of the Heart Institute at the Clinics Hospital of the Medical School of University of São Paulo, Sao Paulo, Brazil; d Department of Cardiac Surgery, Medical University of Vienna, Vienna, Austria; e Leipzig Heart Center, Leipzig–Klinikum Links der Weser, Bremen, Germany; f Department of Cardiac Surgery, Louis Pradel Cardiologic Hospital, Lyon, France; g Division of Cardiac Surgery, IRCCS Azienda Ospedaliero-Universitaria di Bologna, Bologna, Italy; h University of Turin, Turin, Italy; i Department of Cardiac Surgery, Medical Faculty, Heinrich Heine University, Duesseldorf, Germany; j II Department of Anesthesiology, Centre of Anesthesia, Intensive Care and Pain Management, Vilnius University Hospital Santariskiu Klinikos, Vilnius, Lithuania; k Division of Cardiothoracic and Vascular Surgery, Pontchaillou University Hospital, Rennes, France; l Center for Cardiac Intensive Care, Beijing Institute of Heart, Lung, and Blood Vessels Diseases, Beijing Anzhen Hospital, Beijing, China; m Department of Intensive Care Adults, and Department of Cardiology, Thoraxcenter, Erasmus MC, Rotterdam, The Netherlands; n Department of Intensive Care Medicine, Center of Applied Medical Research, St Vincent's Hospital, Darlinghurst, and University of New South Wales, Sidney, Australia; o Department of Cardiology, Fundación Cardiovascular de Colombia, Bucaramanga, Colombia; p Department of Cardiovascular Sciences, University Hospitals Leuven, Leuven, Belgium; q Departments of Medicine and Surgery, University of Maryland, Baltimore, Md; r Cardiochirurgia Ospedali Riuniti “Umberto I Lancisi Salesi” Università Politecnica delle Marche, Ancona, Italy; s Division of Cardiac Surgery, Cardiothoracic Department, University Hospital of Udine, Udine, Italy; t Cardiothoracic Intensive Care Unit, National University Heart Centre, National University Hospital, Singapore; u Cardiac Surgery Unit, Cardiac Thoracic and Vascular Department, Niguarda Hospital, Milan, Italy; v Department of Medicine and Surgery, University of Parma, Cardiac Surgery Unit, University Hospital of Parma, Parma, Italy; w Division of Cardiovascular and Thoracic Surgery, Department of Surgery, Faculty of Medicine Siriraj Hospital, Mahidol University, Bangkok, Thailand; x Department of Cardiothoracic Surgery, University Hospital Henri-Mondor, Créteil, Paris, France; y Department of Cardiothoracic Surgery, University Medical Center Regensburg, Regensburg, Germany; z Department for the Treatment and Study of Cardiothoracic Diseases and Cardiothoracic Transplantation, IRCCS-ISMETT (Istituto Mediterraneo per i Trapianti e Terapie ad Alta Specializzazione); Department of Precision Medicine in Medical Surgical and Critical Area (Me.Pre.C.C.), University of Palermo, Palermo, Italy; aa ECMO Unit, Departamento de Anestesia, Clínica Las Condes, Las Condes, Santiago, Chile; bb Division of Cardiac Surgery, Memorial Healthcare System, Hollywood, Fla; cc Department of Thoracic and Cardiovascular Surgery, Korea University Anam Hospital, Seoul, South Korea; dd Second Department of Internal Medicine, Cardiovascular Medicine General Teaching Hospital and 1st Faculty of Medicine, Charles University in Prague, Prague, Czech Republic; ee Intensive Care Unit, The Alfred Hospital, Melbourne, Victoria, Australia; ff Ospedale del Cuore Fondazione Toscana “G. Monasterio”, Massa, Italy; gg Department of Cardiovascular Surgery, Ziekenhuis Oost-Limburg, Genk, Belgium; hh Cardiac Surgery Unit, IRCCS Humanitas Research Hospital, Rozzano, Italy; ii IU Health Advanced Heart & Lung Care, Indiana University Methodist Hospital, Indianapolis, Ind; jj Adult Intensive Care Services, The Prince Charles Hospital, Brisbane, Australia; kk Cardiac Intensive Care Unit, Johns Hopkins Hospital, Baltimore, Md

**Keywords:** extracorporeal membrane oxygenation, cardiac surgery, cardiogenic shock, complications, emergency, extracorporeal life support

## Abstract

**Background:**

Outcomes in cardiac surgery are influenced by surgical priority, with higher mortality in emergency cases. Whether this applies to postcardiotomy venoarterial (VA) extracorporeal membrane oxygenation (ECMO) remains unknown. This study describes characteristics and outcomes of patients undergoing cardiac operations and requiring VA ECMO, stratified by emergency, urgent, or elective operation.

**Methods:**

This retrospective multicenter observational study included adults requiring postcardiotomy VA ECMO between 2000 and 2020. Preoperative and procedural characteristics, complications, and survival were compared among the 3 patient groups. The association between emergency surgery and in-hospital survival was investigated through mixed Cox proportional hazard models.

**Results:**

The study cohort comprised 1063 patients (52.2%) with elective operations, 445 (21.8%) with urgent operations, and 528 (26%) with emergency operations. Emergency operations included more coronary artery bypass grafting operations (n = 286; 54.2%; *P* < .001) and aortic procedures (n = 126; 23.9%; *P* = .001) in patients with more unstable preoperative hemodynamic conditions compared to elective and urgent patients. VA ECMO was initiated more frequently intraoperatively in emergency patients (n = 353; 66.9%; *P* < .001). Postoperative bleeding (n = 338; 64.3%; *P* < .001), stroke (n = 79; 15%; *P* < .001), and right ventricular failure (n = 124; 25.3%) were more frequent after emergency operations. In-hospital mortality was 60.5% in the elective group, 57.8% in the urgent group, 63.4% in the emergency group (*P* = .191). The crude hazard ratio for in-hospital mortality in emergency surgery was 1.15 (95% confidence interval [CI], 1.01-1.32; *P* = .039) and dropped to 1.09 (95% CI, 0.93-1.27; *P* = .295) after adjustment for indicators of preoperative instability. 5-year survival was comparable in 30-day survivors (*P* = .083).

**Conclusions:**

One-quarter of postcardiotomy VA ECMOs are implemented after emergency operations. Despite more complications in emergency cases, in-hospital and 5-year survival are comparable between emergency, urgent, or elective operations.

## Graphical Abstract

**Figure:**
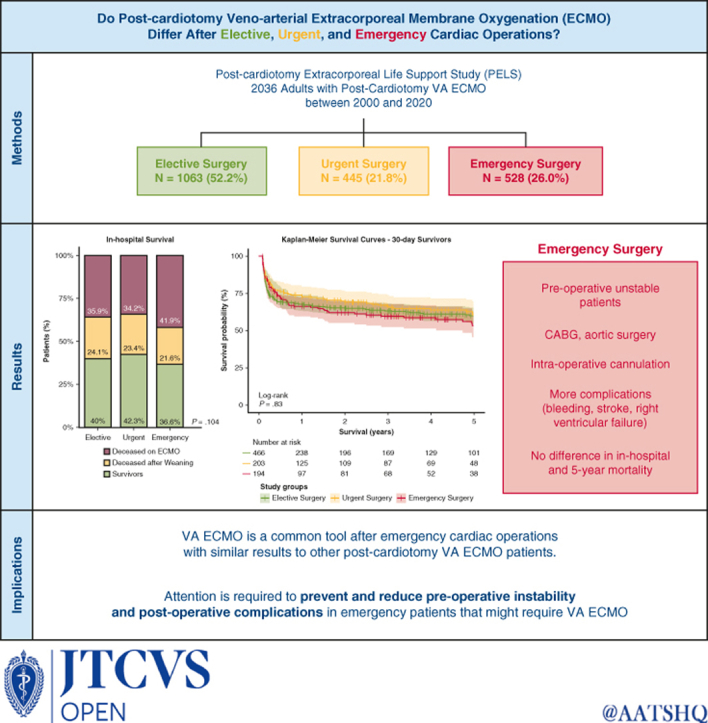

**Figure:**
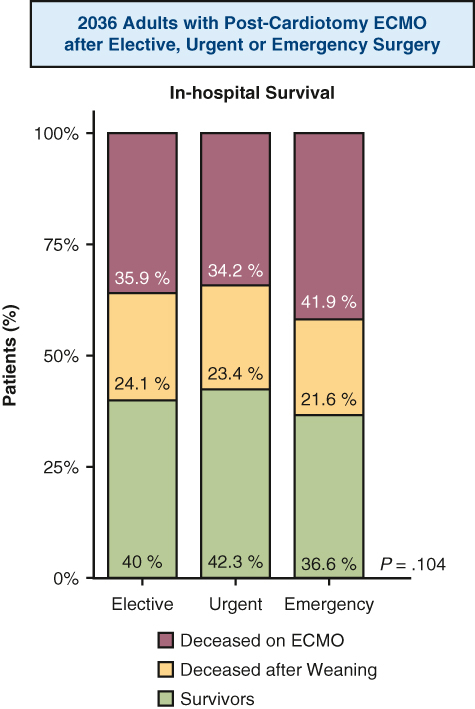
Differences in postcardiotomy ECMO after elective, urgent, or emergency heart operations.

**Central Message**

Profiles of patients requiring extracorporeal membrane oxygenation after elective, urgent, and emergency operations differ. Despite the greater frequency of complications in emergency patients, in-hospital mortality and 5-year survival are similar in the 3 groups of patients.

**Perspective**

This study shows that one-quarter of postcardiotomy extracorporeal membrane oxygenation procedures are implemented after emergency operations, indicating an expansion of indications to more preoperatively unstable patients with a higher prevalence of coronary and aortic diseases. Dedicated strategies are needed to optimize these patients' preoperative critical state and prevent complications to further improve their in-hospital course.

Postoperative outcomes in all surgeries are affected by the urgency or emergency of the operation,^1^ and this has been demonstrated for cardiac surgery as well.^2^^,^^3^ For example, surgical priority is considered a predictor of mortality in the EuroSCORE II^2^ and the Society of Thoracic Surgeons operative risk calculator,^4^ both of which are commonly used to estimate a patient's risk in cardiac surgery. Nevertheless, urgent or emergency procedures remain common, accounting for up to 25% of all cardiac operations.^2^ Among postoperative conditions contributing to these patients' complexity, postcardiotomy cardiogenic shock is associated with a fatal outcome in 50% to 80% of cases.^5^^,^^6^

Venoarterial (VA) extracorporeal membrane oxygenation (ECMO) is often used as support for postcardiotomy shock.^7^^,^^8^ However, based on the higher risk of postoperative complications after urgent or emergency operations,^1^, ^2^, ^3^ it can be speculated that postcardiotomy VA ECMO also might be burdened by an unfavorable risk/benefit ratio in these patients. Thus far, there is little reported data on the relationship between elective, urgent, or emergency operations and postcardiotomy VA ECMO outcomes.^9^ Consequently, clear indications for patient selection based on surgical priority are not available.

This study aimed to describe surgical priority-stratified characteristics, in-hospital outcomes, and survival at follow-up of patients undergoing cardiac surgery who required VA ECMO. We hypothesized that patients requiring VA ECMO after an urgent or emergency procedure would experience more complications and have higher mortality compared to elective surgery patients requiring the same type of circulatory support for postcardiotomy cardiogenic shock.

## Methods

### Study Design

The Postcardiotomy Extracorporeal Life Support Study (PELS) is an international multicenter retrospective observational study collecting data on patients supported with postcardiotomy ECMO (ClinicalTrials.gov identifier NCT03857217).^7^^,^^10^^,^^11^ The study was conducted in accordance with the Declaration of Helsinki. Institutional Review Board approval was obtained at the coordinating center (approval METC-2018-0788; approved December 19, 2018) and was required at all participating centers. The need for informed consent was waived based on the study's retrospective nature, the urgency of the performed procedure, and the deidentification of data. Data are available from the corresponding author on request and with permission from the participating centers.

### Patient Population and Study Groups

**Figure E1:**
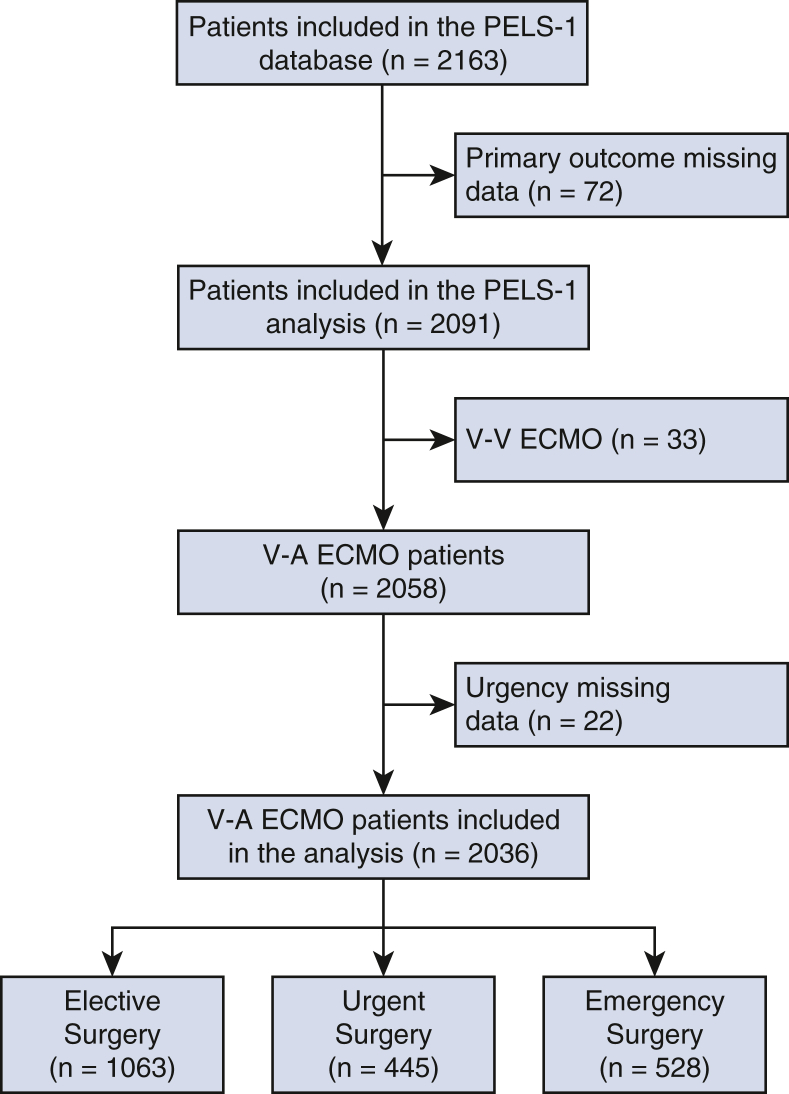
Study flow chart. *PELS*, Postcardiotomy Extracorporeal Life Support; *V-V*, venovenous; *ECMO*, extracorporeal membrane oxygenation; *V-A*, venoarterial.

Adult patients (age ≥18 years) who underwent ECMO during or after their cardiac surgery between January 2000 and December 2020 were included. The exclusion criterion was ECMO support after a noncardiac surgical procedure or unrelated to hospitalization for cardiac surgery. In the present analysis, patients receiving VA ECMO after elective (routine admission for operation), urgent (not admitted electively but needing surgery during the current admission for medical reasons, and cannot be discharged without a definitive procedure), or emergency (operation before the beginning of the next working day after the decision to operate) operations were classified according to the EuroSCORE definitions^2^ (Figure E1).

### Data Collection and Outcomes

Data were included in a dedicated electronic case report form, according to the predefined protocol and variable definitions, as described previously.^7^^,^^10^^,^^11^ The primary outcome of interest was in-hospital mortality, defined as death from any cause. Secondary outcomes included in-hospital complications and 5-year mortality in patients who survived for 30 days after cannulation.^12^

### Statistical Analysis

Data were merged and analyzed using SPSS 26.0 (IBM) and R 4.1.2 (R Foundation for Statistical Computing). Demographic and clinical variables were expressed as number and valid percentage for available data, excluding missing values (Table E1), for categorical variables and as median (interquartile range [IQR]) for continuous variables. No imputations were performed for descriptive statistics. Categorical data were compared among the 3 study groups using the Pearson χ^2^ or Fisher exact test. Continuous variables were analyzed using the independent-samples *t* test or the Kruskal-Wallis test, as appropriate. Post hoc comparisons were performed and adjusted by Bonferroni correction for multiple tests.

The association between emergency surgery and in-hospital mortality was investigated using mixed-effects Cox proportional hazards regression models (Appendix E2) containing both fixed and random effects to account for dependency of observations because of clustering in centers and years. The models were developed on 5 datasets after imputation of variables with <20% missing data. We report measures of association as hazard ratio (HR) with 95% confidence interval (CI).

Based on evidence indicating favorable 5-year survival in patients surviving for 30 days after ECMO cannulation,^12^ the analysis of 5-year survival was performed on 30-day survivors using the Kaplan-Meier method, and comparisons were performed with the log-rank test. Owing to the possible variations in ECMO management over the study period, 2 analyses were performed after stratification of patients who received postcardiotomy ECMO between 2000 and 2010 or between 2011 and 2020. A 2-sided *P* value < .05 was considered statistically significant.

## Results

### Baseline, Surgical, and ECMO Characteristics

The study cohort comprised 2036 patients (Figure E1), stratified into elective (n = 1063; 52.2%), urgent (n = 445; 21.8%), and emergency (n = 528; 26.0%) cases. Preoperative characteristics (Table 1), such as previous or recent myocardial infarction (*P* < .001) and previous percutaneous coronary intervention (*P* < .001), were more frequent in the emergency patient group (Table E2). The emergency group had higher preoperative creatinine levels (*P* < .001) and more frequent cardiogenic shock (*P* < .001), need for mechanical ventilation (*P* < .001) and vasopressors (*P* < .001), and episodes of cardiac arrest (*P* < .001), septic shock (*P* < .001) and acute pulmonary edema (*P* < .001).

**Table 1:** Preoperative characteristics of the study population

Characteristic	Elective surgery (N = 1063)	Urgent surgery (N = 445)	Emergency surgery (N = 528)	*P* value
Age, y, median (IQR)	66.0 (55.6-72.0)	64.0 (55.0-71.0)	64.3 (54.7-72.0)	.215
Sex, n (%)				.074
Female	448 (42.2)	185 (41.6)	192 (36.4)	
Male	614 (57.8)	260 (58.4)	336 (63.6)	
Body mass index, kg/m^2^, median (IQR)	26.2 (23.4-30.0)	26.5 (23.7-29.6)	26.6 (24.2-30.4)	.115
Body surface area, m^2^, median (IQR)	1.9 (1.7-2.0)	1.9 (1.7-2.0)	1.9 (1.8-2.1)	<.001
Comorbidities, n (%)				
Hypertension	668 (65.7)	282 (65.3)	350 (67.8)	.648
Dialysis	73 (7.1)	57 (13.5)	46 (8.9)	<.001
Previous myocardial infarction	202 (19.0)	133 (29.9)	214 (40.5)	<.001
Myocardial infarction (within last 30 d)	51 (5.0)	66 (15.2)	112 (21.7)	<.001
Smoking	232 (24.7)	111 (30.9)	124 (27.9)	.063
Previous stroke	151 (14.2)	59 (13.3)	69 (13.1)	.786
Atrial fibrillation	313 (29.4)	125 (28.1)	99 (18.8)	<.001
Diabetes mellitus	255 (24.0)	125 (28.1)	137 (25.9)	.235
Implanted pacemaker	67 (6.9)	44 (10.9)	25 (5.0)	.002
Implantable cardioverter defibrillator	78 (8.0)	79 (19.4)	25 (5.0)	<.001
Chronic obstructive pulmonary disease	120 (11.6)	46 (10.9)	39 (7.6)	.054
Peripheral artery disease	141 (13.3)	79 (17.8)	76 (14.4)	.078
Chronic pulmonary embolism	34 (3.4)	6 (1.4)	1 (0.2)	<.001
Pulmonary hypertension (>50 mm Hg)	219 (20.7)	101 (22.9)	97 (18.4)	.230
Previous cardiac surgery	265 (24.9)	169 (38.0)	99 (18.8)	<.001
Preoperative creatinine, μmol/L, median (IQR)	96.4 (77.8-123.8)	108.7 (84.0-151.2)	110.4 (87.0-159.2)	<.001
Left ventricular ejection fraction, %, median (IQR)	50 (35-60)	40 (25-58)	40 (25-55)	<.001
EuroSCORE II, median (IQR)	4.2 (2.0-9.7)	12.5 (5.4-25.5)	20.2 (10.8-34.4)	<.001
Preoperative condition, n (%)				
NYHA class				<.001
I	76 (7.4)	32 (7.5)	36 (7.5)	
II	303 (29.6)	64 (15.0)	50 (10.4)	
III	497 (48.5)	148 (34.7)	122 (25.3)	
IV	148 (14.5)	183 (42.9)	275 (56.9)	
Cardiogenic shock	69 (6.5)	85 (19.5)	278 (54.0)	<.001
Intubation	59 (5.6)	45 (10.1)	127 (24.1)	<.001
Cardiac arrest	55 (5.2)	35 (8.0)	97 (18.4)	<.001
Septic shock	9 (0.9)	16 (3.7)	25 (4.9)	<.001
Vasopressors	64 (6.0)	80 (18.2)	168 (32.1)	<.001
Acute pulmonary edema	22 (2.2)	41 (9.6)	77 (15.1)	<.001
Right ventricular failure	64 (6.8)	54 (14.3)	59 (12.4)	<.001
Diagnosis, n (%)				
Coronary artery disease	472 (44.4)	202 (45.4)	305 (57.8)	<.001
Aortic vessel disease	160 (15.1)	60 (13.5)	115 (21.8)	<.001
Aortic valve disease	413 (38.9)	141 (31.7)	137 (25.9)	<.001
Mitral valve disease	388 (36.5)	143 (32.1)	160 (30.3)	.032
Tricuspid valve disease	191 (18.0)	74 (16.6)	61 (11.6)	.004
Post-AMI ventricular septal rupture	10 (0.9)	15 (3.4)	33 (6.3)	<.001
Free wall/papillary muscle rupture	8 (0.8)	5 (1.1)	25 (4.7)	<.001
Active endocarditis	33 (3.1)	57 (12.8)	57 (10.8)	<.001

*IQR*, Interquartile range; *NYHA*, New York Heart Association; *AMI*, acute myocardial infection.

**Figure 1:**
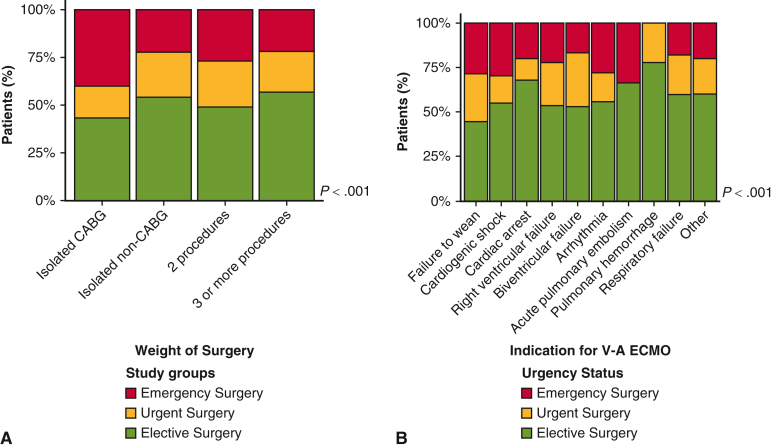
Stacked bar charts representing the distribution of elective, urgent and emergency patients based on weight of surgery (A) and indications for venoarterial extracorporeal membrane oxygenation (*VA ECMO*) initiation (B).

Rates of coronary artery bypass grafting (CABG; *P* < .001), aortic surgery (*P* = .001), pulmonary embolectomy (*P* < .001), and ventricular septal rupture repair (*P* < .001) were higher in the emergency group (Table 2, Figure 1). Intraoperative VA ECMO initiation (*P* < .001) and left ventricular unloading strategies (*P* < .001) also were more frequent in the emergency group. Intra-aortic balloon pump (IABP) use (*P* < .001; Table 3) was higher in emergency patients, especially in the first decade (2000-2010: n = 76, 59.8%; 2011-2020: n/N = 129/401, 32.6%; *P* < .001) (Table E3). The use of an Impella device (n = 9; 0.4%) and other mechanical circulatory support devices (n = 22; 1.1%) were reported in a minority of patients.

**Table 2:** Procedural characteristics

Characteristic	Elective surgery (N = 1063)	Urgent surgery (N = 445)	Emergency surgery (N = 528)	*P* value
Coronary artery bypass graft, n (%)	453 (42.6)	159 (35.7)	286 (54.2)	<.001
Aortic valve, n (%)	411 (38.7)	140 (31.5)	153 (29.0)	<.001
Mitral valve, n (%)	373 (35.1)	136 (30.6)	127 (24.1)	<.001
Tricuspid valve, n (%)	177 (16.7)	60 (13.5)	34 (6.4)	<.001
Aortic vessel, n (%)	185 (17.4)	70 (15.7)	126 (23.9)	.001
Pulmonary valve, n (%)	10 (0.9)	2 (0.4)	0 (0)	.063
Left ventricular assist device, n (%)	9 (0.8)	9 (2.0)	5 (0.9)	.129
Right ventricular assist device, n (%)	0 (0)	4 (0.9)	2 (0.4)	.012
Atrial septal defect repair, n (%)	21 (2.0)	7 (1.6)	10 (1.9)	.869
Ventricular septal defect repair, n (%)	14 (1.3)	21 (4.7)	32 (6.1)	<.001
Ventricular surgery, n (%)	33 (3.1)	17 (3.8)	25 (4.7)	.263
Rhythm surgery, n (%)	50 (4.7)	10 (2.2)	5 (0.9)	<.001
Pulmonary embolectomy, n (%)	4 (0.4)	5 (1.1)	14 (2.7)	<.001
Pulmonary endarterectomy, n (%)	29 (2.7)	7 (1.6)	12 (2.3)	.398
Heart transplantation, n (%)	88 (8.3)	92 (20.7)	25 (4.7)	<.001
Cardiopulmonary bypass time, min, median (IQR)	209 (140-295)	205 (148-281)	193 (131-278)	.107
Cross-clamp time, min, median (IQR)	106 (71-156)	99 (68-138)	80 (49-137)	<.001
Intraoperative transfusions, n (%)	448 (90.0)	174 (94.1)	153 (98.1)	.002

*IQR*, Interquartile range.

**Table 3:** Details of extracorporeal membrane oxygenation

Variable	Elective surgery (N = 1063)	Urgent surgery (N = 445)	Emergency surgery (N = 528)	*P* value
Chest status, n (%)				.015
Chest closed	396 (54.2)	185 (58.4)	273 (62.9)	
Chest open	334 (45.8)	132 (41.6)	161 (37.1)	
ECMO initiation timing, n (%)				<.001
Intraoperative	598 (56.3)	316 (71.0)	353 (66.9)	
Postoperative	465 (43.7)	129 (29.0)	175 (33.1)	
Cannulation approach, n (%)				<.001
Only central cannulation	201 (18.9)	56 (12.6)	84 (15.9)	
Only peripheral cannulation	543 (51.1)	218 (49.0)	203 (38.4)	
Mixed/switch cannulation	291 (27.4)	160 (36.0)	235 (44.5)	
Unknown	28 (2.6)	11 (2.5)	6 (1.1)	
IABP, n (%)	303 (29)	112 (25.2)	205 (39.2)	<.001
IABP positioning, n (%)				<.001
Preoperative	46 (15.2)	48 (42.9)	98 (47.8)	
Intraoperative	257 (84.8)	64 (57.1)	107 (52.2)	
Left ventricular unloading, n (%)	290 (31.6)	90 (24)	138 (37.2)	<.001
ECMO duration, h, median (IQR)	120.0 (67.1-194.7)	106.0 (50.5-169.1)	120.0 (51.4-195)	.031

*ECMO*, Extracorporeal membrane oxygenation; *IABP*, intra-aortic balloon pump.

### In-Hospital Outcomes and Follow-up Survival

**Figure 2:**
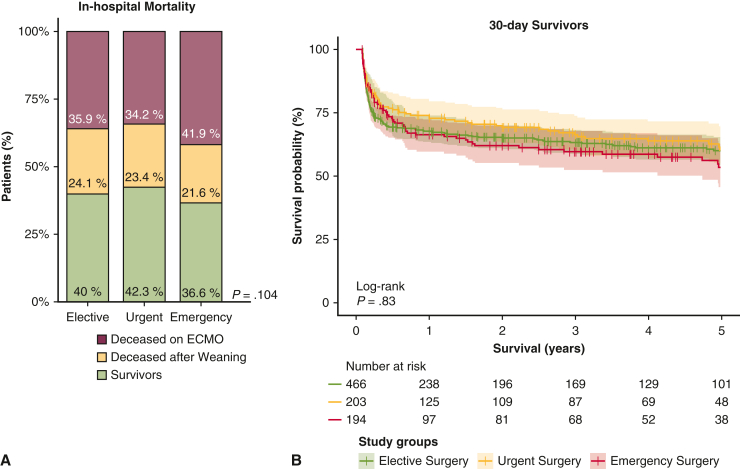
A, In-hospital mortality. B, Kaplan-Meier survival curves with 95% confidence intervals for patients who survived for 30 days after cannulation. *ECMO*, extracorporeal membrane oxygenation.

The emergency group had higher rates of postoperative bleeding (*P* < .001), stroke (*P* < .001), right ventricular failure (*P* < .001), and systemic embolism (*P* < .001). In-hospital survival ranged from 57.8% (n = 257) in the urgent group to 63.4% (n = 335) in the emergency group (*P* = .191) (Table 4, Figure 2). In 30-day survivors, 5-year survival probability was comparable between the groups (*P* = .83).

**Table 4:** Postoperative outcomes

Outcome	Elective surgery (N = 1063)	Urgent surgery (N = 445)	Emergency surgery (N = 528)	*P* value
Intensive care unit stay, d, median (IQR)	14 (6-26)	14 (7-26)	12 (5-25)	.137
Hospital stay, d, median (IQR)	21 (9-40)	21 (9-42)	16 (5-35)	<.001
Postoperative bleeding, n (%)	571 (55.1)	235 (53.8)	338 (64.3)	<.001
Requiring re-thoracotomy	393 (39.6)	159 (38.5)	205 (40.2)	.869
Cannulation site bleeding	95 (9.2)	63 (14.3)	86 (16.3)	<.001
Diffuse non–surgical-related bleeding	219 (23.1)	96 (23.9)	157 (31.7)	.001
Neurologic complications, n (%)				
Cerebral hemorrhage	27 (2.7)	12 (2.8)	25 (4.9)	.061
Severity				.011
Minor	4 (20.0)	4 (44.4)	13 (72.2)	
Disabling	7 (35.0)	4 (44.4)	3 (16.7)	
Fatal	9 (35.0)	1 (11.1)	2 (11.1)	
Seizure	16 (1.6)	12 (2.8)	13 (2.6)	.256
Stroke	93 (8.8)	41 (9.2)	79 (15.0)	<.001
Severity				.006
Minor	28 (35.9)	20 (54.1)	34 (57.6)	
Disabling	26 (33.3)	15 (40.5)	15 (25.4)	
Fatal	24 (30.8)	2 (5.4)	10 (16.9)	
Arrhythmias, n (%)	335 (34.6)	125 (30.6)	158 (31.9)	.293
Leg ischemia, n (%)	90 (9.0)	53 (12.6)	55 (10.8)	.118
Cardiac arrest, n (%)	170 (17.6)	58 (14.2)	74 (14.9)	.204
Pacemaker implant, n (%)	31 (3.2)	12 (2.9)	13 (2.6)	.819
Bowel ischemia, n (%)	42 (4.3)	30 (7.3)	35 (7.1)	.030
Right ventricular failure, n (%)	168 (17.6)	94 (23.6)	124 (25.3)	.001
Acute kidney injury, n (%)	504 (51.7)	259 (63.2)	298 (60.6)	<.001
Pneumonia, n (%)	210 (22.0)	88 (22.2)	111 (22.5)	.978
Septic shock, n (%)	167 (17.5)	71 (17.9)	72 (14.6)	.298
Distributive shock syndrome, n (%)	84 (8.8)	54 (13.6)	38 (7.7)	.006
Acute respiratory distress syndrome, n (%)	50 (5.2)	16 (3.9)	37 (7.5)	.054
Multiorgan failure, n (%)	360 (34.2)	147 (33.7)	187 (36.0)	.721
Embolism, n (%)	38 (4.0)	32 (8.0)	41 (8.4)	<.001
Postoperative procedures, n (%)				
Percutaneous coronary intervention	27 (2.9)	10 (2.6)	11 (2.3)	.824
Cardiac surgery	203 (21.0)	91 (22.2)	116 (23.3)	.582
Abdominal surgery	48 (5.1)	16 (4.2)	21 (4.4)	.730
Vascular surgery	100 (10.6)	60 (15.8)	49 (10.2)	.016
In-hospital mortality, n (%)	643 (60.5)	257 (57.8)	335 (63.4)	.191

*IQR*, Interquartile range.

In mixed-effects Cox models, emergency surgery showed a crude HR of 1.15 (95% CI, 1.01-1.32; *P* = .039) for in-hospital mortality compared to elective/urgent surgery (Tables 5 and E4). The HR dropped to 1.09 (95% CI, 0.93-1.27; *P* = .295) after adjustment for variables reflecting preoperative hemodynamic status and clinical severity (Models 4-7).

**Table 5:** Association between emergency surgery and in-hospital mortality by mixed-effects Cox model with random center and year effects

Model	Emergency surgery	
	HR (95% CI)	*P* value
Model 1: crude model	1.15 (1.01-1.32)	.039
Model 2: Model 1+ sex, age	1.20 (1.05-1.37)	.008
Model 3: Model 2+ preoperative comorbidities	1.28 (1.12-1.47)	<.001
Model 4: Model 2+ preoperative clinical status	1.09 (0.93-1.27)	.295
Model 5: Models 3+ 4+ intraoperative variables	1.08 (0.93-1.27)	.315
Model 6: Model 5+ ECMO details	1.06 (0.90-1.24)	.478
Model 7: Model 6+ postoperative complications	1.04 (0.88-1.22)	.652

For this purpose, elective and urgent surgery were aggregated as reference group. *HR*, Hazard ratio; *CI*, confidence interval; *ECMO*, extracorporeal membrane oxygenation.

### Subgroup Analyses: 2000-2010 and 2011-2020

**Figure E2:**
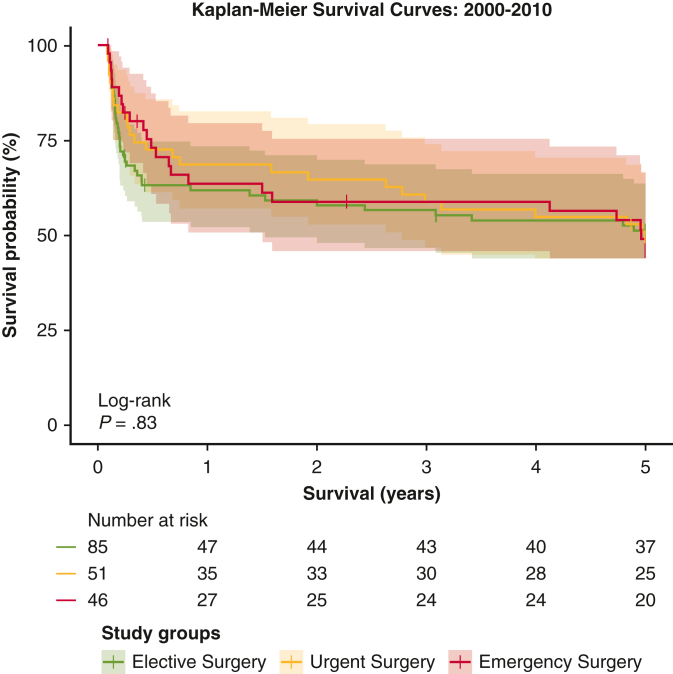
Kaplan-Meier survival curves with 95% confidence intervals for patients who survived for 30 days after cannulation and who received treatment in 2000-2010.

**Figure E3:**
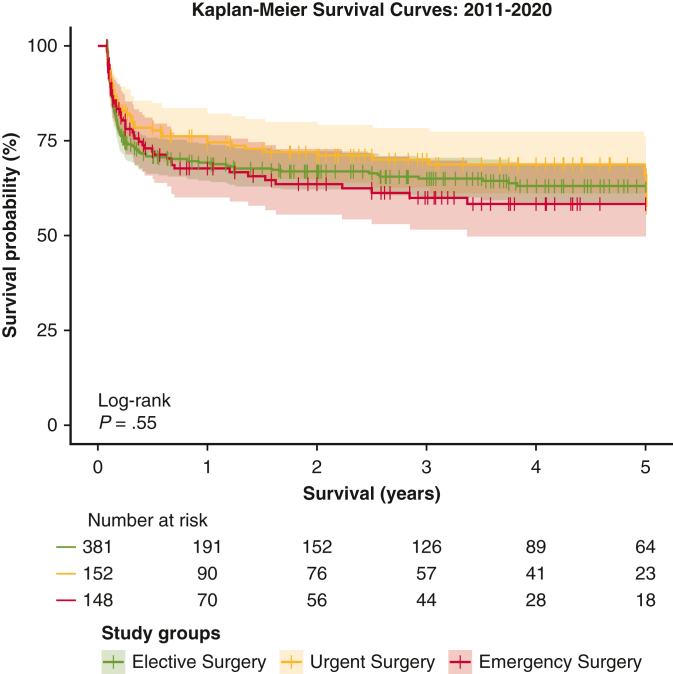
Kaplan-Meier survival curves with 95% confidence intervals for patients who survived for 30 days after cannulation and who received treatment in 2011-2020.

The proportions of elective cases (2000-2010: n = 221/451, 49.0%; 2011-2020: n = 842/1585, 53.1%), urgent cases (2000-2010: n = 103/451, 22.8%; 2011-2020: n = 342/1585, 21.6%), and emergency (2000-2010: n = 127/451, 28.7%; 2011-2020: n = 401/1585, 25.3%) cases were comparable in the 2 subgroups (*P* = .281) (Table E10, Table E11, Table E12, Table E5, Table E6, Table E7, Table E8, Table E9). In the 2000-2010 subgroup, 30.4% (n = 38) of the emergency patients had a preoperative cardiac arrest, although this proportion was 14.7% (n = 59) in the 2011-2020 cohort (*P* < .001). Aortic surgery was performed in 13.4% (n = 17) of cases in the 2000-2010 group and in 27.2% (n = 109) of cases in the 2011-2020 group (*P* = .001). The use of left ventricular unloading was comparable in the 2 groups (*P* = .949). Postoperative bleeding (2000-2010: n = 279, 62.0%; 2011-2020: n = 865, 55.8%; *P* = .020), right ventricular failure (2000-2010: n = 105, 25.7%; 2011-2020: n = 281, 19.6%; *P* = .009), and acute kidney injury (2000-2010: n = 238, 68.9%; 2011-2020: n = 778, 53.1%; *P* < .001) were more frequent in the 2000-2010 group. In-hospital mortality was comparable in the 2 groups (*P* = .190; Figures E2 and E3).

## Discussion

This study demonstrates that VA ECMO support after emergency cardiac operations is common practice. Despite increased complications, in-hospital mortality is only slightly higher than the overall average of 60%, and 5-year survival is comparable to that in urgent or elective cases in 30-day survivors. Key findings include (1) one-quarter of the patients who receive postcardiotomy VA ECMO undergo emergency surgery; (2) VA ECMO after emergency operations is more frequent in patients with vascular diseases requiring coronary artery bypass and aortic procedures, with higher rates of emergency aortic procedures after 2010; (3) emergency patients are characterized by an unstable preoperative hemodynamic profile necessitating more vasopressors and intubation; (4) 11% of urgent patients and 19% of emergency patients requiring VA ECMO are supported with an IABP preoperatively and receive intraoperative VA ECMO; and (5) emergency patients experience more complications, including bleeding, stroke, and right ventricular failure, with comparable 5-year survival in those alive at 30 days after cannulation.

This study shows that emergency patients constitute one-quarter of postcardiotomy VA ECMO cases, highlighting the relevance of this population.^9^ Biancari and colleagues reported 23.7% of emergency cases and 5% of salvage cases among postcardiotomy ECMO patients.^13^ Similarly, an analysis of postcardiotomy VA ECMO runs from the Netherlands Heart Registration database revealed rates of 24.3% in emergency operations and 11.5% in salvage operations.^8^ Nevertheless, although postcardiotomy VA ECMO after emergency or salvage surgery is not rare, information on this group's traits and outcomes is limited, leaving a knowledge gap regarding these patients and their selection and management.

Most emergency patients requiring postcardiotomy VA ECMO suffer from vascular diseases. Approximately 58% of them have coronary artery disease, with a risk profile including prior myocardial infarction and percutaneous coronary interventions. Moreover, 11% of emergency patients are affected by complications of myocardial infarction,^14^^,^^15^ such as free wall or papillary muscle rupture (4.7%) and ventricular septal rupture (6.3%) requiring ventricular septal repair (6.1%). More than one-half (54%) of the emergency patients needing postcardiotomy VA ECMO undergo CABG, as also was observed by Ivanov and colleagues.^9^ In the subgroup of emergency CABG patients, we found that 53% (n = 151/285; data not shown) received an IABP, with 47.7% (n = 72/151) preoperative implants. When considering all patients, 11% (n = 48/445) of urgent cases and 19% (n = 98/428) of emergency cases require IABP preoperatively, and IABP is frequently used as primary unloading strategy across all groups. Despite the consistent use of left ventricular unloading strategies over time, the overall use of IABPs has declined after 2010.^16^ Nevertheless, even with the rise of new unloading strategies, IABPs remain often used in cardiac surgery, particularly for CABG patients.^17^, ^18^, ^19^

Approximately one-quarter (24.0%) of emergency patients required aortic surgery, with a significant increase from a rate of 13.4% in 2000-2010 to 27.2% in 2011-2020. This increase marks the paradigm shift in VA ECMO for aortic patients. Historically, aortic dissections were considered unsuitable for VA ECMO because of bleeding, stroke, and potential vessel damage from VA ECMO flow.^20^ Subsequent research has demonstrated comparable results between patients with and those without dissection,^21^ thus encouraging the use of VA ECMO in this population, as also demonstrated in the present study.

As often happens in coronary or aortic patients requiring emergency operations, the preoperative hemodynamic profile of the emergency cohort is characterized by major instability. Preoperative cardiogenic shock, cardiac arrest, septic shock, and acute pulmonary edema are all more frequent in emergency cases. These conditions may have resulted in higher rates of preoperative intubation and vasopressor use, as well as more frequent intraoperative cannulation, in our emergency cases compared to elective cases.^11^ This peculiar critical state could mediate intrinsic risks in emergency patients.^2^^,^^4^ Indeed, based on the results of the current study, emergency cases have a crude HR of 1.15 (95% CI, 1.01-1.32) for in-hospital mortality. After adjustment by adding the aforementioned indicators of preoperative instability to the mixed Cox model, the HR for emergency surgery decreased to 1.09 (95% CI, 0.93-1.27). These results may suggest that clinicians should focus on preventing or reducing preoperative instability of emergency cases to potentially benefit their in-hospital mortality.

Another target for improvement in emergency patients concerns complications in terms of bleeding (64.3%), stroke (15%), and right ventricular failure (25%). All these events can be expected after aortic operations, which are more frequently associated with bleeding and neurologic events.^21^ Similarly, coronary emergency patients are usually treated with antiplatelet therapies preoperatively,^22^ which carry an increased risk of bleeding during postcardiotomy VA ECMO.^23^ Knowing that these patients represent the largest group of emergency patients requiring postcardiotomy VA ECMO, the current analysis should stimulate the development of ad hoc strategies addressing these issues.

Observed in-hospital mortality rates were 58% in our urgent patients and 63% in our emergency patients, in line with the average 60% reported in most postcardiotomy VA ECMO studies.^6^^,^^13^^,^^24^ This 5% mortality gap in emergency patients supports their candidacy for postcardiotomy VA ECMO. Moreover, this mortality gap seems to become even less relevant when considering the 5-year survival of patients alive at 30 days after cannulation. Such evidence highlights that the initial in-hospital course is the major limiting phase to survival and reinforces the need for strategies to address the aforementioned in-hospital critical phases. Thus, preoperative optimization, prevention of hemodynamic instability, dedicated management strategies for specific patient groups, complication prevention, and close postdischarge follow-up of postcardiotomy ECMO patients should be encouraged to improve outcomes, especially after emergency operations.^8^

### Study Limitations

The structured data collection of the PELS study and the large sample size enhance data robustness and statistical power. However, postcardiotomy ECMO retrospective observational studies, by design, suffer from confounding by indication. Despite this, we adopted a prevalent observational descriptive statistical approach to remain as close as possible to the observed reality. For the current analysis, case classification was based on the EuroSCORE II surgical priority^2^ rather than on specific indications, introducing a selection bias related to local policies and surgical team availability. Differences in ECMO care over the 20-year study period might have added confounding factors; however, to control for these factors, we took center and year effects into account using mixed-effects Cox regression analyses and stratified results by treatment decade. The local policies for left ventricular unloading differed among participating centers, preventing any speculation on relationships between cardiac unloading and enhanced myocardial recovery/ability to wean off VA ECMO support. An in-depth analysis of preoperative, intraoperative, and postoperative hemodynamic parameters; serial arterial lactate concentrations before and during ECMO support; anesthesiology practices; and quality of life, disability, functional status, and rehospitalization after discharge were not possible. The PELS database did not differentiate between emergency and salvage procedures, but these latter patients were included in the emergency group, introducing a possible bias. Finally, we cannot exclude partial overlapping with previously reported series.^25^

## Conclusions

**Figure 3:**
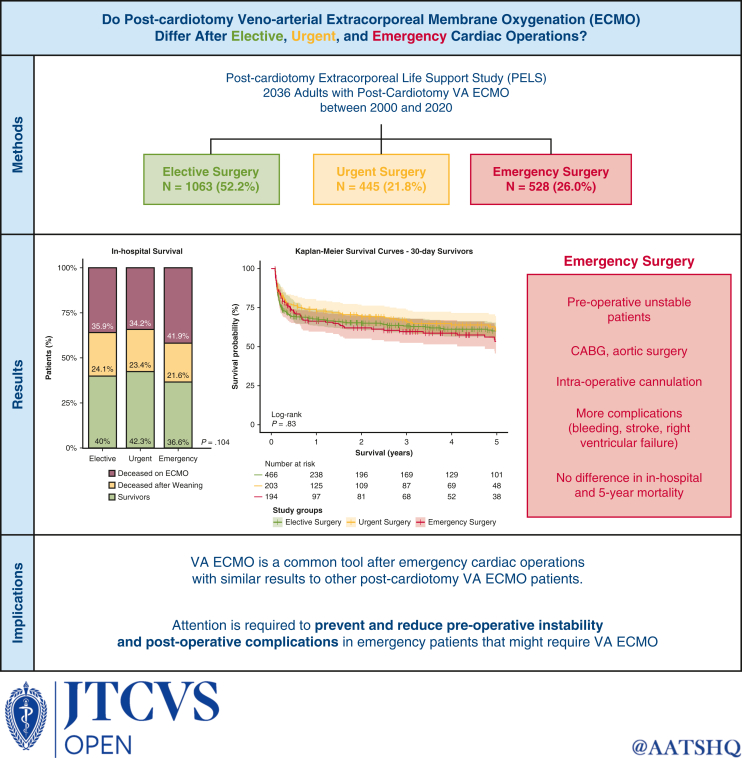
Graphical abstract. Analysis of the Postcardiotomy Extracorporeal Life Support (PELS) study (n = 2036 patients). One-quarter of postcardiotomy venoarterial extracorporeal membrane oxygenation (VA ECMO) procedures are performed after emergency operations in unstable patients undergoing more coronary and aortic operations. Despite more complications, their 63% in-hospital mortality is comparable to that of elective or urgent patients. Patients requiring emergency surgery should not be excluded from postcardiotomy VA ECMO support, but special attention is required to prevent and reduce their preoperative instability and postoperative complications. *CABG*, coronary artery bypass grafting.

Approximately one-quarter of all postcardiotomy VA ECMOs are performed during or after emergency operations, especially in unstable patients with vascular diseases and requiring surgery for CABG, patients with mechanical complications of myocardial infarction, and those undergoing aortic operations (Figure 3). Postcardiotomy VA ECMO after emergency surgery is associated with higher rates of complications, such as bleeding, stroke, and right ventricular failure, and has 63% in-hospital mortality, which is slightly higher but not significantly different than mortality after elective and urgent surgery. For this reason, patients requiring emergency surgery should not be excluded from postcardiotomy VA ECMO support, but special attention is required to prevent and reduce their preoperative instability and postoperative complications to improve their initial in-hospital outcomes.

## Conflict of Interest Statement

Dr Lorusso reported speaker fees from Abiomed, member of the Medical Advisory Board of Xenios and Eurosets, and consultant contract as well as research grants from Medtronic and LivaNova, all honoraria payed to the institution and unrelated to the submitted work. Dr Wiedemann reported consulting/proctoring for Abbott and serving as a scientific advisor for Xenios. All other authors reported no conflicts of interest.

The *Journal* policy requires editors and reviewers to disclose conflicts of interest and to decline handling or reviewing manuscripts for which they may have a conflict of interest. The editors and reviewers of this article have no conflicts of interest.

## Figures and Tables

**Table E1:** Complete and missing cases for each study variable

Variable	Complete cases, n (%)	Missing cases, n (%)
Age (y)	2035 (100)	1 (0)
Sex	2035 (100)	1 (0)
Body mass index (kg/m^2^)	2024 (99.4)	12 (0.6)
Body surface area (m^2^)	2024 (99.4)	12 (0.6)
Comorbidities		
Hypertension	1964 (96.5)	72 (3.5)
Dialysis	1970 (96.8)	66 (3.2)
Previous myocardial infarction	2036 (100)	0 (0)
Myocardial infarction (last 30 d)	1965 (96.6)	71 (3.4)
Smoking	1744 (85.7)	292 (14.3)
Previous stroke	2036 (100)	0 (0)
Atrial fibrillation	2035 (100)	1 (0)
Diabetes mellitus	2036 (100)	0 (0)
Implanted pacemaker	1877 (92.2)	159 (7.8)
Implanted cardioverter-defibrillator	1880 (92.3)	156 (7.7)
Chronic obstructive pulmonary disease	1970 (96.8)	66 (3.2)
Peripheral artery disease	2036 (100)	0 (0)
Chronic pulmonary embolism	1947 (95.6)	89 (4.4)
Pulmonary hypertension (>50 mm Hg)	2023 (99.4)	13 (0.6)
Previous cardiac surgery	2036 (100)	0 (0)
Preoperative creatinine (μmol/L)	1889 (92.8)	147 (7.2)
Left ventricular ejection fraction (%)	1942 (95.4)	94 (4.6)
Euroscore II	1424 (69.9)	612 (30.1)
Preoperative condition		
NYHA class	1934 (94.0)	102 (5.0)
Preoperative cardiogenic shock	2011 (98.8)	25 (1.2)
Preoperative intubation	2036 (100)	0 (0)
Preoperative cardiac arrest	2015 (99.0)	21 (1.0)
Preoperative septic shock	1955 (96.0)	81 (4.0)
Preoperative vasopressors	2025 (99.5)	11 (0.5)
Preoperative acute pulmonary oedema	1953 (95.9)	83 (4.1)
Preoperative right ventricular failure	1793 (88.1)	243 (11.9)
Diagnosis		
Coronary artery disease	2036 (100)	0 (0)
Aortic vessel disease	2036 (100)	0 (0)
Aortic valve disease	2036 (100)	0 (0)
Mitral valve disease	2036 (100)	0 (0)
Tricuspid valve disease	2036 (100)	0 (0)
Post-AMI ventricular septal rupture	2036 (100)	0 (0)
Free wall/papillary muscle rupture	2036 (100)	0 (0)
Active endocarditis	2036 (100)	0 (0)
Coronary artery bypass graft	2036 (100)	0 (0)
Aortic valve surgery	2036 (100)	0 (0)
Mitral valve surgery	2036 (100)	0 (0)
Tricuspid valve surgery	2036 (100)	0 (0)
Aortic surgery	2036 (100)	0 (0)
Pulmonary valve surgery	2036 (100)	0 (0)
Left ventricular assist device	2036 (100)	0 (0)
Right ventricular assist device	2036 (100)	0 (0)
Atrial septal defect repair	2036 (100)	0 (0)
Ventricular septal defect repair	2036 (100)	0 (0)
Ventricular surgery	2036 (100)	0 (0)
Rhythm surgery	2036 (100)	0 (0)
Pulmonary embolectomy	2036 (100)	0 (0)
Pulmonary endarterectomy	2036 (100)	0 (0)
Heart transplantation	2036 (100)	0 (0)
Cardiopulmonary bypass time (min)	1851 (90.9)	185 (9.1)
Cross-clamp time (min)	1838 (90.3)	198 (9.7)
Intraoperative transfusions	839 (41.2)	1197 (58.8)
Extracorporeal life support indication	1989 (97.7)	47 (2.3)
Chest status	1481 (72.2)	555 (27.3)
Initiation timing	2036 (100)	0 (0)
Cannulation approach	2036 (100)	0 (0)
Intra-aortic balloon pump	2012 (98.8)	24 (1.2)
Left ventricular unloading	1663 (81.7)	373 (18.3)
ECMO duration (h)	1999 (98.2)	37 (1.8)
Intensive care unit stay (d)	1951 (95.8)	85 (4.2)
Hospital stays (d)	1963 (96.4)	73 (3.6)
Postoperative bleeding	1999 (98.2)	37 (1.8)
Requiring rethoracotomy	1915 (94.1)	121 (5.9)
Cannulation site bleeding	1999 (98.2)	37 (1.8)
Diffuse non–surgical-related bleeding	1847 (90.7)	189 (9.3)
Neurologic complications		
Cerebral hemorrhage	1930 (94.8)	106 (5.2)
Seizure	1931 (94.8)	105 (5.2)
Stroke	2023 (99.4)	13 (0.6)
Arrhythmia	1872 (91.9)	164 (8.1)
Leg ischemia	1925 (94.5)	111 (5.5)
Cardiac arrest	1874 (92.0)	162 (8.0)
Pacemaker implant	1872 (91.9)	164 (8.1)
Bowel ischemia	1873 (92.0)	163 (8.0)
Right ventricular failure	1845 (90.6)	191 (9.4)
Acute kidney injury	1876 (92.1)	160 (7.9)
Pneumonia	1846 (90.7)	190 (9.3)
Septic shock	1845 (90.6)	191 (9.4)
Distributive shock syndrome	1845 (90.6)	191 (9.4)
Acute respiratory distress syndrome	1872 (91.9)	164 (8.1)
Multiorgan failure	2009 (98.7)	27 (1.3)
Embolism	1845 (90.6)	191 (9.4)
Postoperative procedures		
Percutaneous coronary intervention	1796 (88.2)	240 (11.8)
Cardiac surgery	1875 (92.1)	161 (7.9)
Abdominal surgery	1796 (88.2)	240 (11.8)
Vascular surgery	1800 (88.4)	236 (11.6)
In-hospital mortality	2036 (100)	0 (0)

*NYHA*, New York Heart Association; *AMI*, acute myocardial infarction; *ECMO*, extracorporeal membrane oxygenation.

**Table E2:** Post hoc analyses for significant between-group differences

Variable	Elective surgery (N = 1063)	Urgent surgery (N = 445)	Emergency surgery (N = 528)
	(A)	(B)	(C)
Body surface area		A (.032)	A (<.001)
Dialysis		A (<.001)	
Previous myocardial infarction		A (<.001)	A (<.001) B (.002)
Myocardial infarction (last 30 d)		A (<.001)	A (<.001) B (.033)
Atrial fibrillation	C (<.001)	C (.002)	
Implanted pacemaker		A (.037) C (.002)	
Implanted cardioverter-defibrillator		A (<.001) C (<.001)	
Previous percutaneous coronary intervention			A (<.001) B (.043)
Chronic pulmonary embolism	C (<.001)		
Previous cardiac surgery	C (.017)	A (<.001) C (<.001)	
Preoperative creatinine (μmol/L)		A (<.001)	A (<.001)
Left ventricular ejection fraction (%)	B (<.001) C (<.001)		
EuroSCORE II		A (<.001)	A (<.001) B (<.001)
NYHA class			
I			
II	B (<.001) C (<.001)		
III	B (<.001) C (<.001)	C (.006)	
IV		A (<.001)	A (<.001) B (<.001)
Preoperative cardiogenic shock		A (<.001)	A (<.001) B (<.001)
Preoperative intubation		A (.004)	A (<.001) B (<.001)
Preoperative cardiac arrest			A (<.001) B (<.001)
Preoperative septic shock		A (<.001)	A (<.001)
Preoperative vasopressors		A (<.001)	A (<.001) B (<.001)
Preoperative acute pulmonary oedema		A (<.001)	A (<.001) B (.034)
Preoperative right ventricular failure		A (<.001)	A (.001)
Coronary artery disease			A (<.001) B (<.001)
Aortic vessel disease			A (.002) B (.002)
Aortic valve disease	B (.025) C (<.001)		
Mitral valve disease	C (.043)		
Tricuspid valve disease	C (.003)		
Post-AMI ventricular septal rupture		A (.002)	A (<.001)
Free wall/papillary muscle rupture			A (<.001) B (.004)
Active endocarditis		A (<.001)	A (<.001)
Coronary artery bypass graft	B (.039)		A (<.001) B (<.001)
Aortic valve surgery	B (.024) C (<.001)		
Mitral valve surgery	C (<.001)		
Tricuspid valve surgery	C (<.001)	C (.001)	
Aortic surgery			A (.007) B (.005)
Ventricular septal defect repair		A (<.001)	A (<.001)
Rhythm surgery	C (<0.001)		
Pulmonary embolectomy			A (<.001)
Heart transplantation	C (.029)	A (<.001) C (<.001)	
Cross-clamp time (min)	C (<.001)	C (.032)	
Intraoperative transfusions			A (.004)
Chest status			
Chest open	C (.012)		
Chest closed			A (.012)
ECMO initiation timing			
Intraoperative		A (<.001)	A (<.001)
Postoperative	B (<.001)		
Cannulation approach			
Unknown			
Only central cannulation	B (.009)		
Only peripheral cannulation	C (<.001)	C (.003)	
Mixed/switch cannulation		A (.003)	A (<.001) B (.020)
IABP			A (<.001) B (<.001)
IABP positioning			
Preoperative		A (<.001)	A (<.001)
Intraoperative	B (<.001) C (<.001)		
Left ventricular unloading			A (.001) B (<.001)
Hospital stay		C (.026)	
Postoperative bleeding			A (.002) B (.003)
Cannulation site bleeding		A (.013)	A (<.001)
Diffuse non–surgical-related bleeding			A (.001) B (.032)
Neurologic complications			
Stroke			A (.001) B (.019)
Cerebral hemorrhage severity			
Minor			A (.004)
Disabling			
Fatal			
Stroke severity			
Minor			A (.034)
Disabling			
Fatal	B (.007)		
Right ventricular failure		A (.031)	A (.002)
Acute kidney injury		A (<.001)	A (.004)
Distributive shock syndrome		A (.024) C (.012)	
Embolism		A (.006)	A (.002)
Postoperative procedures			
Vascular surgery		A (.028) C (.045)	

*NYHA*, New York Heart Association; *AMI*, acute myocardial infarction; *ECMO*, extracorporeal membrane oxygenation; *IABP*, intra-aortic balloon pump.

**Table E3:** Additional MCS support in patients treated in 2000-2010 and 2011-2020

Variable	Elective surgery, n (%)	Urgent surgery, n (%)	Emergency surgery, n (%)	*P* value
Patients treated in 2000-2010	N = 221	N = 103	N = 127	
Left ventricular unloading	54 (30.7)	20 (21.5)	39 (42.4)	.009
Left ventricular unloading site				
Right superior pulmonary vein	3 (5.6)	2 (10.0)	2 (5.1)	.009
Left ventricular apex	1 (1.9)	1 (5.0)	4 (10.3)	
Pulmonary artery	1 (1.9)	0 (0)	1 (2.6)	
Septostomy	0 (0)	0 (0)	0 (0)	
Left atrium	17 (31.5)	3 (15)	1 (2.6)	
Transaortic	0 (0)	0 (0)	0 (0)	
Additional venous cannula	0 (0)	0 (0)	0 (0)	
IABP	32 (59.3)	14 (70)	31 (79.5)	
IABP during hospital admission	79 (35.7)	32 (31.1)	76 (59.8)	<.001
IABP positioning				<.001
Preoperative	10 (12.7)	15 (46.9)	43 (56.6)	
Intraoperative	69 (87.3)	17 (53.1)	33 (43.4)	
Impella implantation	1 (0.5)	1 (1.0)	0 (0)	.484
Impella post-ECMO	1 (0,8)	0 (0)	0 (0)	1.0
Other MCS	0 (0)	1 (1.0)	1 (1.0)	.265
Patients treated in 2011-2020	N = 842	N = 342	N = 401	
Left ventricular unloading	236 (31.8)	70 (24.8)	99 (35.5)	.019
Left ventricular unloading site				
Right superior pulmonary vein	18 (7.7)	15 (21.7)	1 (1.0)	.007
Left ventricular apex	13 (5.5)	4 (5.8)	6 (6.1)	
Pulmonary artery	6 (2.6)	4 (5.8)	3 (3.0)	
Septostomy	1 (0.4)	0 (0)	1 (1.0)	
Left atrium	12 (5.1)	1 (1.4)	4 (4.0)	
Transaortic	1 (0.4)	0 (0)	0 (0)	
Additional venous cannula	3 (1.3)	0 (0)	0 (0)	
IABP	181 (77.0)	45 (65.2)	84 (84.4)	
IABP during hospital admission	224 (27.2)	80 (23.5)	129 (32.6)	.02
IABP positioning				<.001
Preoperative	36 (16.1)	33 (41.3)	55 (42.6)	
Intraoperative	188 (83.9)	47 (58.8)	74 (57.4)	
Impella implantation	2 (0.2)	3 (0.9)	2 (0.5)	.317
Impella post-ECMO	1 (0.5)	2 (1.4)	0 (0)	.241
Other MCS	18 (2.3)	1 (0.3)	1 (0.3)	.003

*P* values by the χ^2^ test (for categorical data) indicate statistically significant differences among the 3 groups. *IABP*, Intra-aortic balloon pump; *ECMO*, extracorporeal membrane oxygenation; *MCS*, mechanical circulatory support.

**Table E4:** Association between emergency surgery and in-hospital mortality by mixed-effects Cox models with random center and year effects

Model	HR	95% CI		*P* value
		Lower limit	Upper limit	
Model 1: crude model				
Emergency surgery (reference: elective/urgent)	**1.15**	**1.01**	**1.32**	**.039**
Model 2: Model 1 + sex, age				
Emergency surgery (reference: elective/urgent)	**1.20**	**1.05**	**1.37**	**.008**
Sex (reference: male)	1.10	0.97	1.24	.124
Age	1.02	1.01	1.02	<.001
Model 3. Model 2 + preoperative comorbidities				
Emergency surgery (reference: elective/urgent)	**1.28**	**1.11**	**1.47**	**<.001**
Sex (reference: male)	1.07	0.95	1.21	.239
Age	1.02	1.01	1.02	<.001
Body mass index	1.01	1.00	1.02	.223
Chronic renal failure	1.01	0.82	1.24	.917
Previous myocardial infarction	0.89	0.77	1.02	.103
Previous stroke	1.23	1.02	1.47	.027
Previous atrial fibrillation	1.00	0.88	1.14	.969
Diabetes mellitus	1.01	0.88	1.16	.862
Chronic obstructive pulmonary disease	1.25	1.04	1.50	.018
Peripheral artery disease	1.12	0.95	1.31	.182
Pulmonary hypertension	1.00	0.87	1.16	.985
Previous cardiac surgery	1.04	0.91	1.19	.579
Ischemic ventricular septal rupture	0.99	0.69	1.42	.949
Free wall/papillary muscle rupture	1.16	0.77	1.76	.481
Left ventricular ejection fraction	1.01	1.00	1.01	.004
Model 4: Model 2 + preoperative clinical status				
Emergency surgery (reference: elective/urgent)	**1.09**	**0.93**	**1.27**	**.295**
Sex (reference: male)	1.08	0.96	1.22	.205
Age	1.02	1.01	1.02	<.001
Body mass index	1.01	1.00	1.02	.171
Left ventricular ejection fraction	1.01	1.00	1.01	.001
Preoperative cardiogenic shock	1.16	0.96	1.40	.115
Preoperative cardiac arrest	1.31	1.07	1.61	.008
Preoperative septic shock	1.40	0.97	2.03	.073
Preoperative right ventricular failure	1.22	0.99	1.51	.061
Preoperative intubation	1.12	0.90	1.40	.319
Preoperative IABP	0.97	0.79	1.19	.753
Preoperative vasopressors	1.04	0.85	1.27	.697
Model 5: Models 3 + 4 + intraoperative variables				
Emergency surgery (reference: elective/urgent)	**1.08**	**0.93**	**1.27**	**.315**
Sex (reference: male)	1.09	0.96	1.23	.170
Age	1.02	1.01	1.02	<.001
Body mass index	1.01	0.99	1.02	.319
Chronic renal failure	0.99	0.80	1.22	.917
Previous myocardial infarction	0.95	0.81	1.10	.483
Previous stroke	1.21	1.00	1.45	.044
Previous atrial fibrillation	0.97	0.85	1.12	.693
Diabetes mellitus	1.04	0.90	1.19	.606
Chronic obstructive pulmonary disease	1.27	1.06	1.54	.012
Peripheral artery disease	1.13	0.96	1.34	.130
Pulmonary hypertension	0.98	0.84	1.15	.831
Previous cardiac surgery	0.95	0.83	1.10	.526
Ischemic ventricular septal rupture	1.01	0.69	1.48	.966
Free wall/papillary muscle rupture	1.10	0.71	1.70	.662
Left ventricular ejection fraction	1.00	1.00	1.01	.054
Preoperative cardiogenic shock	1.20	0.99	1.45	.058
Preoperative cardiac arrest	1.41	1.15	1.74	.001
Preoperative septic shock	1.38	0.94	2.04	.102
Preoperative intubation	1.14	0.91	1.42	.252
Preoperative IABP	1.07	0.86	1.33	.547
Preoperative vasopressors	1.05	0.86	1.29	.612
Preoperative right ventricular failure	1.29	1.03	1.61	.026
Ischemic ventricular septal rupture	1.00	1.00	1.00	.008
Coronary artery bypass surgery	1.00	0.88	1.14	.982
Aortic valve surgery	1.17	1.02	1.33	.023
Mitral valve surgery	1.06	0.92	1.23	.398
Tricuspid valve surgery	1.25	1.04	1.50	.020
Aortic surgery	1.34	1.14	1.59	.001
Model 6: model 5 + ECMO details				
Emergency surgery (reference: elective/urgent)	**1.06**	**0.90**	**1.24**	**.478**
Sex (reference: male)	1.09	0.97	1.23	.162
Age	1.02	1.01	1.02	<.001
Body mass index	1.01	0.99	1.02	.364
Chronic renal failure	0.98	0.79	1.21	.840
Previous myocardial infarction	0.96	0.82	1.12	.600
Previous stroke	1.20	1.00	1.44	.050
Previous atrial fibrillation	0.97	0.85	1.11	.673
Diabetes mellitus	1.03	0.90	1.19	.660
Chronic obstructive pulmonary disease	1.28	1.06	1.54	.011
Peripheral artery disease	1.12	0.95	1.32	.174
Pulmonary hypertension	0.99	0.85	1.16	.939
Previous cardiac surgery	0.94	0.82	1.09	.429
Ischemic ventricular septal rupture	1.02	0.70	1.50	.904
Free wall/papillary muscle rupture	1.11	0.72	1.71	.630
Left ventricular ejection fraction	1.00	1.00	1.01	.023
Preoperative cardiogenic shock	1.21	1.00	1.46	.050
Preoperative cardiac arrest	1.37	1.11	1.70	.004
Preoperative septic shock	1.39	0.95	2.05	.090
Preoperative intubation	1.15	0.92	1.43	.231
Preoperative IABP	1.05	0.84	1.31	.691
Preoperative vasopressors	1.07	0.87	1.31	.526
Preoperative right ventricular failure	1.32	1.05	1.67	.018
Cardiopulmonary bypass time (min)	1.00	1.00	1.00	.002
Coronary artery bypass surgery	1.00	0.87	1.14	.966
Aortic valve surgery	1.15	1.01	1.32	.032
Mitral valve surgery	1.03	0.89	1.19	.713
Tricuspid valve surgery	1.26	1.04	1.52	.016
Aortic surgery	1.33	1.13	1.58	.001
ECMO initiation timing (reference: intraoperative)	1.17	1.00	1.38	.049
ECMO indication (reference: failure to wean)				
Acute pulmonary embolism	0.69	0.16	2.88	.606
Arrhythmia	0.57	0.35	0.94	.028
Cardiac arrest	0.97	0.75	1.24	.800
Cardiogenic shock	0.89	0.74	1.08	.247
Pulmonary hemorrhage	0.35	0.11	1.12	.077
Right ventricular failure	0.75	0.60	0.94	.013
Respiratory failure	0.79	0.56	1.11	.173
Biventricular failure	1.04	0.81	1.33	.774
Other	0.50	0.28	0.90	.020
Model 7: Model 6 + postoperative complications				
Emergency surgery (reference: elective/urgent)	**1.04**	**0.88**	**1.22**	**.652**
Sex (reference: male)	1.11	0.98	1.26	.090
Age	1.02	1.01	1.02	<.001
Body mass index	1.00	0.99	1.01	.625
Chronic renal failure	1.01	0.82	1.25	.896
Previous myocardial infarction	0.95	0.81	1.11	.530
Previous stroke	1.15	0.95	1.38	.144
Previous atrial fibrillation	0.98	0.86	1.13	.828
Diabetes mellitus	0.94	0.81	1.08	.359
Chronic obstructive pulmonary disease	1.13	0.93	1.36	.213
Peripheral artery disease	1.18	1.00	1.40	.045
Pulmonary hypertension	0.96	0.82	1.12	.607
Previous cardiac surgery	0.94	0.81	1.08	.372
Ischemic ventricular septal rupture	1.00	0.67	1.48	.994
Free wall/papillary muscle rupture	0.89	0.58	1.38	.614
Left ventricular ejection fraction	1.00	1.00	1.01	.108
Preoperative cardiogenic shock	1.22	1.00	1.48	.047
Preoperative cardiac arrest	1.34	1.08	1.66	.008
Preoperative septic shock	1.42	0.97	2.08	.071
Preoperative intubation	1.12	0.89	1.40	.346
Preoperative IABP	1.05	0.84	1.32	.645
Preoperative vasopressors	1.04	0.84	1.28	.702
Preoperative right ventricular failure	1.11	0.90	1.38	.335
Cardiopulmonary bypass time (min)	1.00	1.00	1.00	.074
Coronary artery bypass surgery	1.04	0.91	1.19	.571
Aortic valve surgery	1.05	0.92	1.20	.440
Mitral valve surgery	0.96	0.84	1.11	.606
Tricuspid valve surgery	1.28	1.05	1.54	.013
Aortic surgery	1.40	1.18	1.66	<.001
ECMO initiation timing (reference: intraoperative)				
ECMO indication (reference: failure to wean)	1.06	0.90	1.25	.494
Acute pulmonary embolism	0.73	0.10	5.53	.746
Arrhythmia	0.66	0.40	1.10	.110
Cardiac arrest	0.87	0.68	1.13	.303
Cardiogenic shock	0.88	0.72	1.06	.185
Pulmonary hemorrhage	0.35	0.11	1.14	.081
Right ventricular failure	0.70	0.56	0.87	.002
Respiratory failure	0.72	0.51	1.03	.076
Biventricular failure	0.94	0.73	1.21	.622
Other	0.45	0.25	0.82	.009
Complications				
Bleeding requiring re-thoracotomy	0.93	0.83	1.06	.271
Cerebral hemorrhage	1.04	0.76	1.44	.786
Stroke	0.79	0.64	0.97	.022
Leg ischemia	1.02	0.85	1.23	.820
Cardiac arrest	1.44	1.23	1.68	<.001
Bowel ischemia	1.10	0.88	1.37	.399
Acute kidney injury	0.92	0.79	1.06	.240
Septic shock	0.68	0.57	0.80	<.001
Multiorgan failure	3.40	2.97	3.91	<.001
Right ventricular failure	1.45	1.24	1.69	<.001

Bold type indicates significance. *HR*, Hazard ratio; *CI*, confidence interval; *IABP*, intra-aortic balloon pump; *ECMO*, extracorporeal membrane oxygenation.

**Table E5:** Preoperative characteristics of patients treated in 2000-2010

Characteristic	Elective surgery (N = 221)	Urgent surgery (N = 103)	Emergency surgery (N = 127)	*P* value
Age, y, median (IQR)	65.7 (56-71)	64.0 (55-69.4)	63.0 (53.9-72)	.547
Sex, n (%)				.284
Female	111 (50.2)	51 (49.5)	53 (41.7)	
Male	110 (49.8)	52 (50.5)	74 (58.3)	
Body mass index, kg/m^2^, median (IQR)	26.1 (23.2-29.7)	26.6 (23.7-31.2)	27.5 (24.5-30.5)	.088
Body surface area, m^2^, median (IQR)	1.8 (1.7-2)	1.9 (1.7-2)	1.9 (1.8-2.1)	<.001
Comorbidities, n (%)				
Hypertension	135 (62.8)	63 (61.8)	77 (60.6)	.923
Dialysis	13 (6.1)	8 (8.6)	10 (8.7)	.605
Previous myocardial infarction	43 (19.5)	31 (30.1)	53 (41.7)	<.001
Myocardial infarction (last 30 d)	5 (2.3)	15 (14.7)	38 (29.9)	<.001
Smoking	46 (25.4)	20 (33.9)	36 (40.4)	.037
Previous stroke	35 (15.8)	14 (13.6)	19 (15)	.870
Atrial fibrillation	63 (28.5)	33 (32.0)	26 (20.5)	.115
Diabetes mellitus	60 (27.1)	22 (21.4)	38 (29.9)	.333
Implanted pacemaker	11 (5.4)	13 (13.7)	6 (4.9)	.018
Implantable cardioverter defibrillator	21 (10.2)	15 (15.5)	7 (5.7)	.060
Chronic obstructive pulmonary disease	32 (14.9)	17 (17.9)	12 (10.3)	.281
Peripheral artery disease	40 (18.1)	16 (15.5)	24 (18.9)	.787
Previous transplant	5 (2.3)	6 (5.9)	3 (2.4)	.199
Chronic pulmonary embolism	12 (5.7)	1 (1.1)	0 (0)	.007
Pulmonary hypertension (>50 mm Hg)	54 (24.5)	31 (30.7)	25 (20.0)	.179
Previous cardiac surgery	57 (25.8)	44 (42.7)	28 (22.0)	.001
Preoperative creatinine, μmol/L, median (IQR)	95.0 (79.6-123.8)	114.9 (97.2-161.8)	114.1 (89.0-173.3)	<.001
Left ventricular ejection fraction, %, median (IQR)	52 (35-60)	35 (21-55)	40 (25-54)	<.001
EuroSCORE II, median (IQR)	3.9 (2.0-8.7)	12.0 (4.4-29.4)	28.7 (18.1-47.1)	<.001
Preoperative condition, n (%)				
NYHA class				<.001
I	13 (6.1)	4 (4.0)	6 (5.2)	
II	67 (31.5)	9 (9.0)	10 (8.6)	
III	107 (50.2)	36 (36.0)	26 (22.4)	
IV	26 (12.2)	51 (51.0)	74 (63.8)	
Cardiogenic shock	4 (1.8)	16 (15.8)	66 (55.5)	<.001
Intubation	1 (0.5)	10 (9.7)	37 (29.1)	<.001
Cardiac arrest	18 (8.2)	10 (9.9)	38 (30.4)	<.001
Septic shock	0 (0)	2 (2.0)	3 (2.4)	.083
Vasopressors	2 (0.9)	22 (22.2)	48 (39.0)	<.001
Acute pulmonary edema	5 (2.3)	10 (10.2)	21 (17.2)	<.001
Right ventricular failure	19 (10.2)	14 (20.0)	12 (11.9)	.103
Diagnosis				
Coronary artery disease	92 (41.6)	42 (40.8)	76 (59.8)	.002
Aortic vessel disease	22 (10.0)	11 (10.7)	16 (12.6)	.746
Aortic valve disease	93 (42.1)	34 (33.0)	31 (24.4)	.004
Mitral valve disease	71 (32.1)	33 (32.0)	42 (33.1)	.980
Tricuspid valve disease	32 (14.5)	13 (12.6)	8 (6.3)	.071
Post-AMI ventricular septal rupture	2 (0.9)	3 (2.9)	9 (7.1)	.006
Free wall/papillary muscle rupture	2 (0.9)	1 (1.0)	8 (6.3)	.004
Active endocarditis	2 (0.9)	8 (7.8)	8 (6.3)	.004

*IQR*, Interquartile range; *NYHA*, New York Heart Association; *AMI*, acute myocardial infarction.

**Table E6:** Procedural characteristics of patients treated in 2000-2010

Characteristic	Elective surgery (N = 221)	Urgent surgery (N = 103)	Emergency surgery (N = 127)	*P* value
Coronary artery bypass graft, n (%)	84 (38.0)	37 (35.9)	72 (56.7)	<.001
Aortic valve surgery, n (%)	87 (39.4)	32 (31.1)	31 (24.4)	.015
Mitral valve surgery, n (%)	69 (31.2)	32 (31.1)	32 (25.2)	.457
Tricuspid valve surgery, n (%)	26 (11.8)	16 (15.5)	4 (3.1)	.005
Aortic surgery, n (%)	28 (12.7)	12 (11.7)	17 (13.4)	.925
Pulmonary valve surgery, n (%)	1 (0.5)	0 (0)	0 (0)	.594
Left ventricular assist device, n (%)	0 (0)	1 (1.0)	2 (1.6)	.200
Right ventricular assist device, n (%)	0 (0)	1 (1.0)	1 (0.8)	.373
Atrial septal defect repair, n (%)	3 (1.4)	1 (1.0)	2 (1.6)	.923
Ventricular septal defect repair, n (%)	3 (1.4)	5 (4.9)	8 (6.3)	.040
Ventricular surgery, n (%)	5 (2.3)	2 (1.9)	2 (1.6)	.906
Rhythm surgery, n (%)	9 (4.1)	0 (0)	0 (0)	.008
Pulmonary embolectomy, n (%)	1 (0.5)	2 (1.9)	3 (2.4)	.270
Pulmonary endarterectomy, n (%)	13 (5.9)	3 (2.9)	3 (2.4)	.219
Heart transplantation, n (%)	33 (14.9)	28 (27.2)	8 (6.3)	<.001
Cardiopulmonary bypass time, min, median (IQR)	207 (136-294)	219 (149-293)	190 (135-283)	.279
Cross-clamp time, min, median (IQR)	102 (69-158)	91 (67-125)	61 (34-114)	<.001
Intraoperative transfusions, n (%)	66 (85.7)	15 (93.8)	15 (93.8)	.499

*IQR*, Interquartile range.

**Table E7:** ECMO in patients treated in 2000-2010

Variable	Elective surgery (N = 221)	Urgent surgery (N = 103)	Emergency surgery (N = 127)	*P* value
Chest status, n (%)				.125
Chest closed	88 (53.7)	59 (66.3)	67 (61.5)	
Chest open	76 (46.3)	30 (33.7)	42 (38.5)	
ECMO initiation timing, n (%)				<.001
Intraoperative	121 (54.8)	81 (78.6)	94 (74.0)	
Postoperative	100 (45.2)	22 (21.4)	33 (26.0)	
Cannulation approach, n (%)				.079
Only central cannulation	53 (24.0)	15 (14.6)	28 (22)	
Only peripheral cannulation	114 (51.6)	59 (57.3)	56 (44.1)	
Mixed/switch cannulation	48 (21.7)	26 (25.2)	40 (31.5)	
Unknown	6 (2.7)	3 (2.9)	3 (2.4)	
ECMO duration, h, median (IQR)	116.0 (56.7-192.0)	99.5 (48.5-157)	86.4 (44.0-134.3)	.012

*ECMO*, Extracorporeal membrane oxygenation; *IQR*, interquartile range.

**Table E8:** Postoperative outcomes of patients treated in 2000-2010

Outcome	Elective surgery (N = 221)	Urgent surgery (N = 103)	Emergency surgery (N = 127)	*P* value
ICU stay, d, median (IQR)	15 (7-29)	18 (9-28)	10 (4-21)	.002
Hospital stay, d, median (IQR)	20 (8-41)	24 (10-40)	12 (4-30)	.004
Postoperative bleeding, n (%)	140 (63.6)	57 (55.3)	82 (64.6)	.280
Requiring re-thoracotomy	96 (45.5)	42 (44.2)	52 (43.7)	.946
Cannulation site bleeding	20 (9.1)	14 (13.6)	22 (17.3)	.078
Diffuse non–surgical-related bleeding	69 (33.3)	27 (29.0)	44 (37.0)	.477
Neurologic complications, n (%)				
Cerebral hemorrhage	5 (2.4)	2 (2.0)	10 (8.1)	.017
Seizure	5 (2.4)	1 (1.0)	7 (5.8)	.088
Stroke	18 (8.2)	7 (6.8)	18 (14.2)	.106
Arrhythmia, n (%)	90 (44.3)	19 (20.7)	39 (34.2)	<.001
Leg ischemia, n (%)	30 (14.4)	13 (13.4)	18 (14.6)	.962
Cardiac arrest, n (%)	43 (21.2)	9 (9.8)	24 (20.9)	.049
Pacemaker implantation, n (%)	6 (3.0)	2 (2.2)	3 (2.6)	.927
Bowel ischemia, n (%)	8 (3.9)	7 (7.5)	6 (5.3)	.429
Right ventricular failure, n (%)	52 (25.6)	25 (27.2)	28 (24.6)	.913
Acute kidney injury, n (%)	139 (67.5)	60 (65.9)	84 (73.7)	.410
Pneumonia, n (%)	46 (22.7)	21 (22.8)	27 (23.7)	.978
Septic shock, n (%)	39 (19.2)	13 (14.1)	15 (13.2)	.302
Distributive shock syndrome, n (%)	12 (5.9)	4 (4.3)	3 (2.6)	.407
Acute respiratory distress syndrome, n (%)	18 (8.9)	6 (6.5)	12 (10.5)	.601
Multiorgan failure, n (%)	87 (40.1)	32 (33.3)	42 (34.7)	.425
Embolism, n (%)	11 (5.4)	7 (7.6)	12 (10.5)	.245
Postoperative procedures, n (%)				
Percutaneous coronary intervention	2 (1.0)	1 (1.3)	0 (0)	.547
Cardiac surgery	33 (16.2)	7 (7.6)	16 (13.9)	.138
Abdominal surgery	14 (7.1)	3 (3.8)	3 (2.8)	.228
Vascular surgery	21 (10.7)	5 (6.3)	11 (10.2)	.531
In-hospital mortality, n (%)	142 (64.3)	60 (58.3)	84 (66.1)	.437

*IQR*, Interquartile range.

**Table E9:** Preoperative characteristics of patients who received treatment in 2011-2020

Characteristic	Elective surgery (N = 842)	Urgent surgery (N = 342)	Emergency surgery (N = 401)	*P* value
Age, y, median (IQR)	66.0 (55.3-72.0)	64.0 (55.0-72.0)	64.6 (55.0-72.0)	.392
Sex, n (%)				.179
Female	337 (40.1)	134 (39.2)	139 (34.7)	
Male	504 (59.9)	208 (60.8)	262 (65.3)	
Body mass index, kg/m^2^, median (IQR)	26.3 (23.5-30)	26.3 (23.7-29.4)	26.5 (23.9-30.4)	.467
Body surface area, m^2^, median (IQR)	1.9 (1.7-2.0)	1.9 (1.8-2.1)	1.9 (1.8-2.1)	<.001
Comorbidities, n (%)				
Hypertension	533 (66.5)	219 (66.4)	273 (70.2)	.407
Dialysis	60 (7.3)	49 (14.9)	36 (9.0)	<.001
Previous myocardial infarction	159 (18.9)	102 (29.8)	161 (40.1)	<.001
Myocardial infarction (last 30 d)	46 (5.7)	51 (15.4)	74 (19.0)	<.001
Smoking	186 (24.5)	91 (30.3)	88 (24.7)	.129
Previous stroke	116 (13.8)	45 (13.2)	50 (12.5)	.814
Atrial fibrillation	250 (29.7)	92 (26.9)	73 (18.3)	<.001
Diabetes mellitus	195 (23.2)	103 (30.1)	99 (24.7)	.043
Implanted pacemaker	56 (7.3)	31 (10.1)	19 (5.0)	.037
Implantable cardioverter-defibrillator	57 (7.5)	64 (20.6)	18 (4.7)	<.001
Chronic obstructive pulmonary disease	88 (10.7)	29 (8.9)	27 (6.8)	.091
Peripheral artery disease	101 (12)	63 (18.4)	52 (13.0)	.013
Chronic pulmonary embolism	22 (2.8)	5 (1.5)	1 (0.3)	.010
Pulmonary hypertension (>50 mm Hg)	165 (19.7)	70 (20.6)	72 (18.0)	.639
Previous cardiac surgery, n (%)	208 (24.7)	125 (36.5)	71 (17.7)	<.001
Preoperative creatinine, μmol/L, median (IQR)	96.6 (77.0-123.8)	107.9 (80.4-150.0)	110.4 (85.5-154.7)	<.001
Left ventricular ejection fraction, %, median (IQR)	50 (37-60)	40 (25-60)	43 (27-55)	<.001
EuroSCORE II, median (IQR)	4.2 (2.0-10.0)	12.9 (5.5-24.4)	18.5 (8.9-29.0)	<.001
Preoperative condition, n (%)				
NYHA class				<.001
I	63 (7.8)	28 (8.6)	30 (8.2)	
II	236 (29.1)	55 (16.8)	40 (10.9)	
III	390 (48.1)	112 (34.3)	96 (26.2)	
IV	122 (15)	132 (40.4)	201 (54.8)	
Cardiogenic shock	65 (7.7)	69 (20.7)	212 (53.5)	<.001
Intubation	58 (6.9)	35 (10.2)	90 (22.4)	<.001
Cardiac arrest	37 (4.4)	25 (7.4)	59 (14.7)	<.001
Septic shock	9 (1.1)	14 (4.2)	22 (5.7)	<.001
Vasopressors	62 (7.4)	58 (17.0)	120 (29.9)	<.001
Acute pulmonary oedema	17 (2.1)	31 (9.4)	56 (14.4)	<.001
Right ventricular failure	45 (6.0)	40 (13.0)	47 (12.6)	<.001
Diagnosis, n (%)				
Coronary artery disease	380 (45.1)	160 (46.8)	229 (57.1)	<.001
Aortic vessel disease	138 (16.4)	49 (14.3)	99 (24.7)	<.001
Aortic valve disease	320 (38.0)	107 (31.3)	106 (26.4)	<.001
Mitral valve disease	317 (37.6)	110 (32.2)	118 (29.4)	.011
Tricuspid valve disease	159 (18.9)	61 (17.8)	53 (13.2)	.044
Post-AMI ventricular septal rupture	8 (1.0)	12 (3.5)	24 (6.0)	<.001
Free wall/papillary muscle rupture	6 (0.7)	4 (1.2)	17 (4.2)	<.001
Active endocarditis	31 (3.7)	49 (14.3)	49 (12.2)	<.001

*IQR*, Interquartile range; *NYHA*, New York Heart Association; *AMI*, acute myocardial infarction.

**Table E10:** Procedural characteristics of patients treated in 2011-2020

Characteristic	Elective surgery (N = 842)	Urgent surgery (N = 342)	Emergency surgery (N = 401)	*P* value
Coronary artery bypass graft, n (%)	369 (43.8)	122 (35.7)	214 (53.4)	<.001
Aortic valve surgery, n (%)	324 (38.5)	108 (31.6)	122 (30.4)	.007
Mitral valve surgery, n (%)	304 (36.1)	104 (30.4)	95 (23.8)	<.001
Tricuspid valve surgery, n (%)	151 (17.9)	44 (12.9)	30 (7.5)	<.001
Aortic surgery, n (%)	157 (18.6)	58 (17.0)	109 (27.2)	.001
Pulmonary valve surgery, n (%)	9 (1.1)	2 (0.6)	0 (0)	.101
Left ventricular assist device, n (%)	9 (1.1)	8 (2.3)	3 (0.7)	.117
Right ventricular assist device, n (%)	0 (0)	3 (0.9)	1 (0.2)	.024
Atrial septal defect repair, n (%)	18 (2.1)	6 (1.8)	8 (2.0)	.913
Ventricular septal defect repair, n (%)	11 (1.3)	16 (4.7)	24 (6.0)	<.001
Ventricular surgery, n (%)	28 (3.3)	15 (4.4)	23 (5.7)	.135
Rhythm surgery, n (%)	41 (4.9)	10 (2.9)	5 (1.2)	.004
Pulmonary embolectomy, n (%)	3 (0.4)	3 (0.9)	11 (2.7)	<.001
Pulmonary endarterectomy, n (%)	16 (1.9)	4 (1.2)	9 (2.2)	.539
Heart transplantation, n (%)	55 (6.5)	64 (18.7)	17 (4.2)	<.001
Cardiopulmonary bypass time, min, median (IQR)	209 (140-296)	198 (145-279)	193 (128-275)	.166
Cross-clamp time, min, median (IQR)	107 (73-155)	102 (68-142)	86 (55-147)	<.001
Intraoperative transfusions, n (%)	382 (90.7)	159 (94.1)	138 (98.6)	.006

*IQR*, Interquartile range.

**Table E11:** ECMO in patients treated in 2011-2020

Variable	Elective surgery (N = 842)	Urgent surgery (N = 342)	Emergency surgery (N = 401)	*P* value
Chest status, n (%)				.027
Chest closed	308 (54.4)	126 (55.3)	206 (63.4)	
Chest open	258 (45.6)	102 (44.7)	119 (36.6)	
ECMO initiation timing, n (%)				<.001
Intraoperative	447 (56.7)	235 (68.7)	259 (64.6)	
Postoperative	365 (43.3)	107 (31.3)	142 (35.4)	
Cannulation approach, n (%)				<.001
Only central cannulation	148 (17.6)	41 (12.0)	56 (14.0)	
Only peripheral cannulation	429 (51.0)	159 (46.5)	147 (36.7)	
Mixed/switch cannulation	243 (28.9)	134 (39.2)	195 (48.6)	
Unknown	22 (2.6)	8 (2.3)	3 (0.7)	
ECMO duration, h, median (IQR)	120.0 (69.0-196.0)	110.0 (50.8-187.3)	123.1 (59.5-216.0)	.035

*ECMO*, Extracorporeal membrane oxygenation; *IQR*, interquartile range.

**Table E12:** Postoperative outcomes of patients treated in 2011-2020

Outcome	Elective surgery (N = 842)	Urgent surgery (N = 342)	Emergency surgery (N = 401)	*P* value
Intensive care unit stay, d, median (IQR)	13 (6-25)	13 (6-25)	13 (5-27)	.816
Hospital stays, d, median (IQR)	22 (10-40)	20 (9-44)	17 (5-35)	.009
Postoperative bleeding, n (%)	431 (52.8)	178 (53.3)	256 (64.2)	<.001
Requiring re-thoracotomy	297 (38.0)	117 (36.8)	153 (39.1)	.816
Cannulation site bleeding	75 (9.2)	49 (14.5)	64 (16.0)	<.001
Diffuse non–surgical-related bleeding	150 (20.2)	69 (22.4)	113 (30.0)	.001
Neurologic complications, n (%)				
Cerebral hemorrhage	22 (2.8)	10 (3.1)	15 (3.9)	.596
Seizure	11 (1.4)	11 (3.4)	6 (1.6)	.078
Stroke	75 (9.0)	34 (10.0)	61 (15.3)	.003
Arrhythmia, n (%)	245 (32)	106 (33.5)	119 (31.2)	.794
Leg ischemia, n (%)	60 (7.6)	40 (12.3)	37 (9.6)	.043
Cardiac arrest, n (%)	127 (16.6)	49 (15.5)	50 (13.1)	.300
Pacemaker implantation, n (%)	25 (3.3)	10 (3.2)	10 (2.6)	.829
Bowel ischemia, n (%)	34 (4.5)	23 (7.3)	29 (7.6)	.052
Right ventricular failure, n (%)	116 (15.4)	69 (22.5)	96 (25.5)	<.001
Acute kidney injury, n (%)	365 (47.5)	199 (62.4)	214 (56.6)	<.001
Pneumonia, n (%)	164 (21.8)	67 (22.0)	84 (22.1)	.993
Septic shock, n (%)	128 (17)	58 (19.0)	57 (15.0)	.376
Distributive shock syndrome, n (%)	72 (9.6)	50 (16.4)	35 (9.2)	.003
Acute respiratory distress syndrome, n (%)	32 (4.2)	10 (3.2)	25 (6.5)	.078
Multiorgan failure, n (%)	273 (32.7)	115 (33.8)	145 (36.3)	.441
Embolism, n (%)	27 (3.6)	25 (8.2)	29 (7.7)	.002
Postoperative procedures, n (%)				
Percutaneous coronary intervention	25 (3.4)	9 (3.0)	11 (3.0)	.919
Cardiac surgery	170 (22.3)	84 (26.5)	100 (26.1)	.196
Abdominal surgery	34 (4.6)	13 (4.3)	18 (4.9)	.944
Vascular surgery	79 (10.6)	55 (18.3)	38 (10.2)	.001
In-hospital mortality, n (%)	501 (59.5)	197 (57.6)	251 (62.6)	.365

*IQR*, Interquartile range.

## Appendix 1 PELS Investigators

Silvia Mariani,^a,b^ Alvaro Perazzo,^a,c^ Maria Elena De Piero,^a^ Bas C. T. van Bussel,^d^ Samuel Heuts,^a^ Justine Ravaux,^a^ Michele Di Mauro,^a^ Dominik Wiedemann,^e^ Anne-Kristin Schaefer,^e^ Luca Conci,^e^ Diyar Saeed,^f^ Jawad Khalil,^f^ Sven Lehmann,^f^ Matteo Pozzi,^g^ Jean-Francois Obadia,^g^ Antonio Loforte,^h,i^ Luca Botta,^h^ Davide Pacini,^h^ Udo Boeken,^j^ Nikolaos Kalampokas,^j^ Robertas Samalavicius,^k^ Agne Jankuviene,^k^ Karl Bounader,^l^ Erwan Flecher,^l^ Xiaotong Hou,^m^ Jeroen J. H. Bunge,^n,o^ Dinis Dos Reis Miranda,^o^ Hergen Buscher,^p^ Kogulan Sriranjan,^p^ Leonardo Salazar,^q^ Bart Meyns,^r^ Daniel Herr,^s^ Michael A. Mazzeffi,^s^ Sacha Matteucci,^t^ Marco Di Eusanio,^t^ Sandro Sponga,^u^ Igor Vendramin,^u^ Graeme MacLaren,^v^ Vitaly Sorokin,^v^ Kollengode Ramanathan,^v^ Claudio Russo,^w^ Alessandro Costetti,^w^ Francesco Formica,^b,x^ Giovanni Marchetto,^b^ Pranya Sakiyalak,^y^ Antonio Fiore,^z^ Daniele Camboni,^aa^ Chistof Schmid,^aa^ Giuseppe Maria Raffa,^bb^ Rodrigo Diaz,^cc^ Roberto Castillo,^cc^ I-wen Wang,^dd^ Jae-Seung Jung,^ee^ Jan Belohlavek,^ff^ Tomas Grus,^gg^ Vin Pellegrino,^hh^ Giacomo Bianchi,^ii^ Marco Solinas,^ii^ Matteo Pettinari,^jj^ Alessandro Barbone,^kk^ José P. Garcia,^ll^ Kiran Shekar,^mm^ Glenn Whitman,^nn^ and Roberto Lorusso^a^

^a^Cardio-Thoracic Surgery Department, and Cardiovascular Research Institute Maastricht, Maastricht, The Netherlands

^b^Cardiac Surgery Unit, San Gerardo Hospital, Monza, Italy

^c^Transplant Center of the Heart Institute at the Clinics Hospital of the Medical School of University of São Paulo, Sao Paulo, Brazil

^d^Department of Intensive Care Medicine, and Cardiovascular Research Institute Maastricht, Maastricht, The Netherlands

^e^Department of Cardiac Surgery, Medical University of Vienna, Vienna, Austria

^f^Department of Cardiac Surgery, Leipzig Heart Center, Leipzig, Germany

^g^Department of Cardiac Surgery, Louis Pradel Cardiologic Hospital, Lyon, France

^h^Division of Cardiac Surgery, IRCCS Azienda Ospedaliero-Universitaria di Bologna, Bologna, Italy

^i^University of Turin, Turin, Italy

^j^Department of Cardiac Surgery, Medical Faculty, Heinrich Heine University, Duesseldorf, Germany

^k^II Department of Anesthesiology, Centre of Anesthesia, Intensive Care and Pain management, Vilnius University Hospital Santariskiu Klinikos, Vilnius, Lithuania

^l^Division of Cardiothoracic and Vascular Surgery, Pontchaillou University Hospital, Rennes, France

^m^Center for Cardiac Intensive Care, Beijing Institute of Heart, Lung, and Blood Vessels Diseases, Beijing Anzhen Hospital, Capital Medical University, Beijing, China

^n^Department of Cardiology, Erasmus MC, Rotterdam, The Netherlands

^o^Department of Intensive Care Adults, Erasmus MC, Rotterdam, The Netherlands

^p^Department of Intensive Care Medicine, Center of Applied Medical Research, St Vincent's Hospital, Darlinghurst, New South Wales, Australia

^q^Department of Cardiology, Fundación Cardiovascular de Colombia, Bucaramanga, Colombia

^r^Department of Cardiac Surgery, University Hospitals Leuven and Department of Cardiovascular Sciences, University of Leuven, Leuven, Belgium

^s^Departments of Medicine and Surgery, University of Maryland, Baltimore, Md

^t^SOD Cardiochirurgia Ospedali Riuniti “Umberto I Lancisi Salesi” Università Politecnica delle Marche, Ancona, Italy

^u^Division of Cardiac Surgery, Cardiothoracic Department, University Hospital of Udine, Udine, Italy

^v^Cardiothoracic Intensive Care Unit, National University Heart Centre, National University Hospital, Singapore, Singapore

^w^Cardiac Surgery Unit, Cardiac Thoracic and Vascular Department, Niguarda Hospital, Milan, Italy

^x^Department of Medicine and Surgery, University of Parma, Parma, Italy

^y^Division of Cardiovascular and Thoracic Surgery, Department of Surgery, Faculty of Medicine Siriraj Hospital, Mahidol University, Bangkok, Thailand

^z^Department of Cardio-Thoracic Surgery, University Hospital Henri-Mondor, Créteil, Paris, France

^aa^Department of Cardiothoracic Surgery, University Medical Center Regensburg, Regensburg, Germany

^bb^Department for the Treatment and Study of Cardiothoracic Diseases and Cardiothoracic Transplantation, IRCCS-ISMETT (Istituto Mediterraneo per i Trapianti e Terapie ad Alta Specializzazione), Palermo, Italy

^cc^ECMO Unit, Departamento de Anestesia, Clínica Las Condes, Las Condes, Santiago, Chile

^dd^Division of Cardiac Surgery, Memorial Healthcare System, Hollywood, Fla

^ee^Department of Thoracic and Cardiovascular Surgery, Korea University Anam Hospital, Seoul, South Korea

^ff^Second Department of Internal Medicine, Cardiovascular Medicine General Teaching Hospital and 1st Faculty of Medicine, Charles University in Prague, Prague, Czech Republic

^gg^Second Department of Cardiovascular Surgery, First Faculty of Medicine, Charles University and General University Hospital in Prague, Prague, Czech Republic

^hh^Intensive Care Unit, The Alfred Hospital, Melbourne, Victoria, Australia

^ii^Ospedale del Cuore Fondazione Toscana “G. Monasterio,” Massa, Italy

^jj^Department of Cardiovascular Surgery, Ziekenhuis Oost-Limburg, Genk, Belgium

^kk^Cardiac Surgery Unit, IRCCS Humanitas Research Hospital, Rozzano, Italy

^ll^IU Health Advanced Heart & Lung Care, Indiana University Methodist Hospital, Indianapolis, Ind

^mm^Adult Intensive Care Services, The Prince Charles Hospital, Brisbane, Australia

^nn^Cardiac Intensive Care Unit, Johns Hopkins Hospital, Baltimore, Md.

## Appendix 2 Statistical Analysis

Data were merged and analyzed using SPSS 26.0 (IBM), and R 4.1.2 (R Foundation for Statistical Computing). The full cohort was divided into 3 study groups according to the EuroSCORE definitions^E1^ for comparison: elective (patients routinely admitted for operation), urgent (patients not electively admitted but who require surgery during the current admission for medical reasons and cannot be discharged without a definitive procedure) or emergency (patients requiring operation before the beginning of the next working day after the decision to operate). Missing data analysis was conducted with the *mice: Multivariate Imputation by Chained Equations* R package.^E2^ The percentage of missing values was calculated for each variable (Table E1). Missing data patterns were investigated and were identified as missing completely at random.

Descriptive statistics were analyzed on available data only, and no imputations were performed for this purpose. Normality was investigated with the Kolmogorov-Smirnov test, Shapiro-Wilk test, and inspection of histograms and Q-Q plots as appropriate. Homogeneity of variance was tested with the Levene test. Demographic and clinical variables are expressed as number (percentage) for categorical variables. For each 2 × 2 table, the χ^2^ test was used if all expected cell counts were ≥5, otherwise the Fisher exact test was used. The Fisher-Freeman-Halton exact test for tables with more than 2 × 2 cells were used. Continuous variables that complied with normality are reported as mean (SD). If equal variances could be assumed, they were tested by the independent Student *t* test; otherwise the Welch test was used. Values that did not comply with normality are reported as median (interquartile range [IQR]) and tested with the Mann-Whitney *U* test.

Stacked bar plots were designed to represent the distributions of levels within each categorical variable and compare them among the 3 study groups. We described the population characteristics and preoperative variables, intraoperative variables, variables while on extracorporeal membrane oxygenation (ECMO) and postoperative complications stratified for elective, urgent, and emergency operations.

To estimate the associations between surgical priority (reference group: elective/urgent operations) and in-hospital mortality, we conducted a mixed-effects Cox proportional hazards regression, using the *coxme: Mixed Effects Cox Models* R package. The random effect was used to account for the dependency of observations due to clustering in centers and in years. The proportional hazards assumption was tested with Schoenfeld residuals, and nonlinearity for continuous variables was tested with Martingale residuals. We first estimated a crude model, which was adjusted for age and sex, and subsequently for sets of variables comsidered potential confounders of the association with mortality at patient selection, intraoperative decisions, and ECMO management, based on clinical practice and the literature.E3, E4, E5, E6, E7 Forward or backward selection of variables based on statistical arguments were not applied. Each variable set was developed based on clinical practice and the literature.^E8^ For the associations with in-hospital mortality, we used the following models:

Model 1: crude model.
Model 2: demographic data (sex, age).
Model 3: demographic data, body mass index, left ventricular ejection fraction and comorbidities (chronic renal failure, previous myocardial infarction, previous stroke, previous atrial fibrillation, diabetes mellitus, chronic obstructive pulmonary disease, peripheral artery disease, pulmonary hypertension, previous cardiac surgery, ischaemic ventricular septal rupture, free wall/papillary muscle rupture).
Model 4: demographic data, body mass index, left ventricular ejection fraction, and preoperative status (cardiogenic shock, cardiac arrest, septic shock, right ventricular failure, intubation, intra-aortic balloon pump, vasopressors).
Model 5: demographic data, body mass index, left ventricular ejection fraction, comorbidities, preoperative status, and surgical details (cardiopulmonary bypass time, coronary artery bypass surgery, aortic valve surgery, mitral valve surgery, tricuspid valve surgery, aortic surgery).
Model 6: demographic data, body mass index, left ventricular ejection fraction, comorbidities, preoperative status, surgical details, and ECMO details (initiation timing, indication).
Model 7: demographic data, body mass index, left ventricular ejection fraction, comorbidities, preoperative status, surgical details, ECMO details, and complications (bleeding requiring rethoracotomy, cerebral haemorrhage, stroke, leg ischemia, cardiac arrest, bowel ischemia, acute kidney injury, septic shock, multiorgan failure, right ventricular failure).

The mixed-effects Cox proportional hazards models were developed on 5 imputed datasets after imputation of variables with <20% missing data. Variables with more missing data were omitted from the models. We used the *mice: Multivariate Imputation by Chained Equations* R package^E2^ for the imputation process. Five imputed datasets were created with the “cart” method, mixed-Cox models were run on each of these datasets, and results were pooled (*junkka/ehahelper: Helper Functions for Event History Analysis* R package) to obtain estimates as hazard ratios (HRs) with their 95% confidence intervals (CIs) and *P* values.

Survival was investigated with the Kaplan-Meier method, and comparisons were performed with the log-rank test (*survival* and *survminer* R packages). Patient lost to follow-up were included in survival analyses and were considered censored at the time of their last control. Curves were truncated when the number of patients at risk from the study groups dropped below 10% of the initial sample (at 5 years).

Based on the possible variations in ECMO management over the study period, 2 subgroup analyses were performed in patients who received a postcardiotomy ECMO between 2011 and 2020, and in patients who received postcardiotomy ECMO between 2000 and 2010. A 2-sided *P* value < .05 and a *P* value for interaction <.1 were considered to indicate statistically significance.
